# Genome-wide methylation and transcriptome analysis in penile carcinoma: uncovering new molecular markers

**DOI:** 10.1186/s13148-015-0082-4

**Published:** 2015-04-18

**Authors:** Hellen Kuasne, Ilce Mara de Syllos Cólus, Ariane Fidelis Busso, Hector Hernandez-Vargas, Mateus Camargo Barros-Filho, Fabio Albuquerque Marchi, Cristovam Scapulatempo-Neto, Eliney Ferreira Faria, Ademar Lopes, Gustavo Cardoso Guimarães, Zdenko Herceg, Silvia Regina Rogatto

**Affiliations:** CIPE - International Research Center, AC Camargo Cancer Center, Rua Taguá, 440, CEP: 01508-010, Liberdade, São Paulo, SP Brazil; Department of Biology, Londrina State University, Londrina, PR Brazil; Epigenetics Group, International Agency for Research on Cancer (IARC), Lyon, France; Inter-institutional Grad Program on Bioinformatics, Institute of Mathematics and Statistics, USP, São Paulo, SP Brazil; Department of Pathology, CPOM - Molecular Oncology Research Center, Barretos Cancer Hospital, Barretos, SP Brazil; Department of Urology, Barretos Cancer Hospital, Barretos, SP Brazil; Department of Pelvic Surgery, AC Camargo Cancer Center, São Paulo, Brazil; Department of Urology, Faculty of Medicine, UNESP, Botucatu, SP Brazil

**Keywords:** Penile carcinomas, DNA methylome, Human papillomavirus, Molecular marker, Transcriptome

## Abstract

**Background:**

Despite penile carcinoma (PeCa) being a relatively rare neoplasm, it remains an important public health issue for poor and developing countries. Contrary to most tumors, limited data are available for markers that are capable of assisting in diagnosis, prognosis, and treatment of PeCa. We aimed to identify molecular markers for PeCa by evaluating their epigenomic and transcriptome profiles and comparing them with surrounding non-malignant tissue (SNT) and normal glans (NG).

**Results:**

Genome-wide methylation analysis revealed 171 hypermethylated probes in PeCa. Transcriptome profiling presented 2,883 underexpressed and 1,378 overexpressed genes. Integrative analysis revealed a panel of 54 genes with an inverse correlation between methylation and gene expression levels. Distinct methylome and transcriptome patterns were found for human papillomavirus (HPV)-positive (38.6%) and negative tumors. Interestingly, grade 3 tumors showed a distinct methylation profile when compared to grade 1. In addition, univariate analysis revealed that low *BDNF* methylation was associated with lymph node metastasis and shorter disease-free survival. CpG hypermethylation and gene underexpression were confirmed for a panel of genes, including *TWIST1*, *RSOP2*, *SOX3*, *SOX17*, *PROM1*, *OTX2*, *HOXA3*, and *MEIS1*.

**Conclusions:**

A unique methylome signature was found for PeCa compared to SNT, with aberrant DNA methylation appearing to modulate the expression of specific genes. This study describes new pathways with the potential to regulate penile carcinogenesis, including stem cell regulatory pathways and markers associated to a worse prognosis. These findings may be instrumental in the discovery and application of new genetic and epigenetic biomarkers in PeCa.

**Electronic supplementary material:**

The online version of this article (doi:10.1186/s13148-015-0082-4) contains supplementary material, which is available to authorized users.

## Background

Penile carcinoma (PeCa) is a rare neoplasm in developed countries, with an incidence of 0.5 to 1.1 per 100,000 in Europe [1]. However, it represents an important public health problem for poor and developing countries, such as in Brazil, where its incidence varies from 2.9 to 6.8 cases per 100,000 [2]. In India, Africa, and other regions of South America, it is responsible for up to 10% of malignancies that affect men [3]. The etiology and various aspects of PeCa pathophysiology are still poorly understood, with poor penile hygiene and the presence of phimosis being the most strongly associated risk factors [4]. The incidence of the disease differs more than 40-fold in countries where neonatal circumcision and appropriate hygiene are common practice [5]. Other risk factors, such as the number of sexual partners, a history of genital warts and/or other sexually transmitted diseases, balanitis xerotic obliterans, chronic lichen planus, smoking, and zoophilia, have also been described [6,7]. Human papillomavirus (HPV) infection is considered to be a risk factor for a subset of PeCa, although its role in penile carcinogenesis has not been clarified [8-10].

PeCa has an unpredictable outcome, and its current treatment requires partial or total penile amputation. There are few molecular studies on PeCa, with the majority of them having focused on protein expression levels or genetic/epigenetic alterations of specific genes [11,12]. DNA methylation, histone modification, and regulation by non-coding RNA regulate gene expression by controlling chromatin accessibility and transcription [13]. Alterations of epigenetic markers have been associated with the development and progression of various tumors [14]. In PeCa, one of the most studied epigenetic alterations is the hypermethylation of the *CDKN2A* gene promoter, which has been found in 15% to 42% of samples [15]. To date, only one study has analyzed the transcriptome [16], while another has evaluated the epigenetic profile in PeCa [17]. Kroon et al. [16] evaluated 56 PeCa samples using oligoarray analysis and found a 44-probe classifier that predicted lymph node metastasis. However, validation analysis in an independent set of samples failed to confirm it as useful to predict nodal metastases in PeCa. More recently, Feber et al. [17] used high-density genome-wide methylation array to evaluate the methylation profile of 38 PeCa samples and identified epi-signatures related to HPV infection and lymph node metastasis. However, none of these studies have evaluated the impact of DNA methylation on gene expression using large-scale analysis. In the present study, integrated transcriptome and methylome data were used to identify novel epigenetically regulated transcripts with a potential clinical application.

## Results

### Genome-wide methylation, transcriptome, and integrative analysis

**Figure 1:**
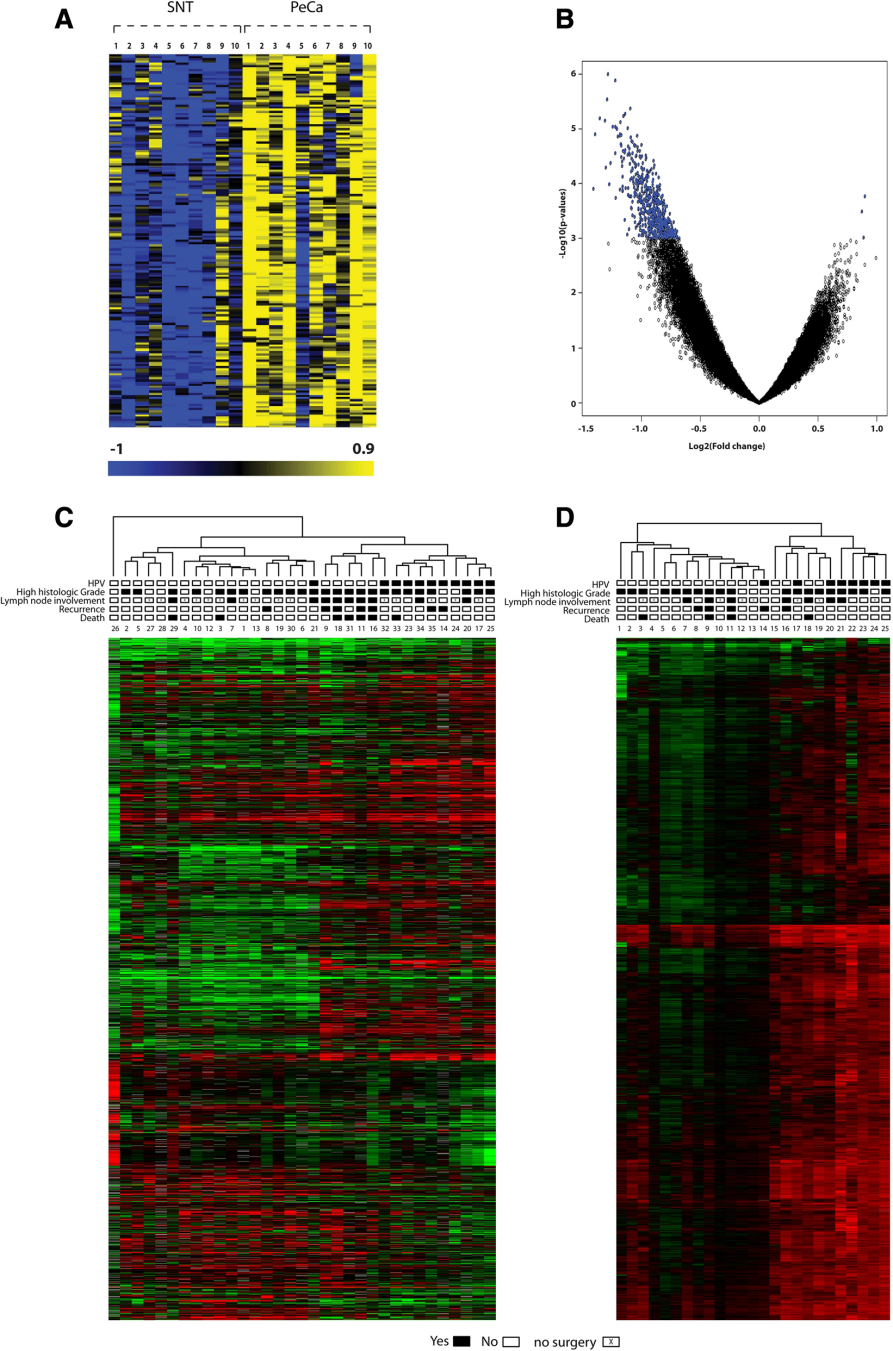
Supervised and unsupervised analysis of gene expression and methylation profiles. **(A)** Heat map showing 171 significantly hypermethylated probes in the paired analysis of 10 PeCa and SNT samples (*P* value ≤0.001 and FDR ≤0.05). **(B)** Volcano plot of the methylated regions: left-sided deviation indicates that all hypermethylated probes were tumor related. **(C)** Unsupervised analysis showing dendogram and heat map of altered transcripts from the gene expression analysis in PeCa. **(D)** Unsupervised analysis showing dendogram and heat map of altered probes from the methylation analysis in 25 PeCa samples. Rectangles show clinical characteristics of patients with PeCa. Numbers below rectangles represent samples.

The methylation profile of 25 PeCa, 10 surrounding non-malignant tissues (SNT), and 4 normal glans (NG) samples was evaluated using methyl-CpG immunoprecipitation microarray (MCIp-chip) (Additional file 1: Figure S1). Reproducibility of the MCIp-chip experiments was controlled by using technical replicates of one randomly selected case (Pearson correlation, *r*^2^ = 0.93 *P* value ≤ 2.2e^−16^). Microarray analysis revealed similar methylome profiles for a pool of four NG and SNT samples, confirming that SNT could be used as a control in the PeCa analysis (data not shown). Using 10 paired PeCa and SNT samples, 171 hypermethylated probes were identified (false discovery rate (FDR) ≤ 0.05; *P* value ≤ 0.001) (Figure 1A, B), which represented 106 annotated genes (Additional file 2: Table S1). Ninety-two percent of the differentially methylated probes were located within promoter regions. In addition, pyrosequencing revealed a global hypomethylation in PeCa samples when compared to NG and SNT, with an average methyl-cytosine loss of 15% in ALR1Sat and AluYB8 sequences (Additional file 1: Figure S2).

Transcriptome analysis was performed on 33 PeCa samples, revealing 3,637 underexpressed (2,883 annotated genes) and 1,730 overexpressed probes (1,378 genes). Interestingly, six members of the matrix metalloproteinase gene family (MMPs) were detected among the 30 genes with a higher fold change in PeCa (Additional file 2: Table S2).

**Figure 2:**
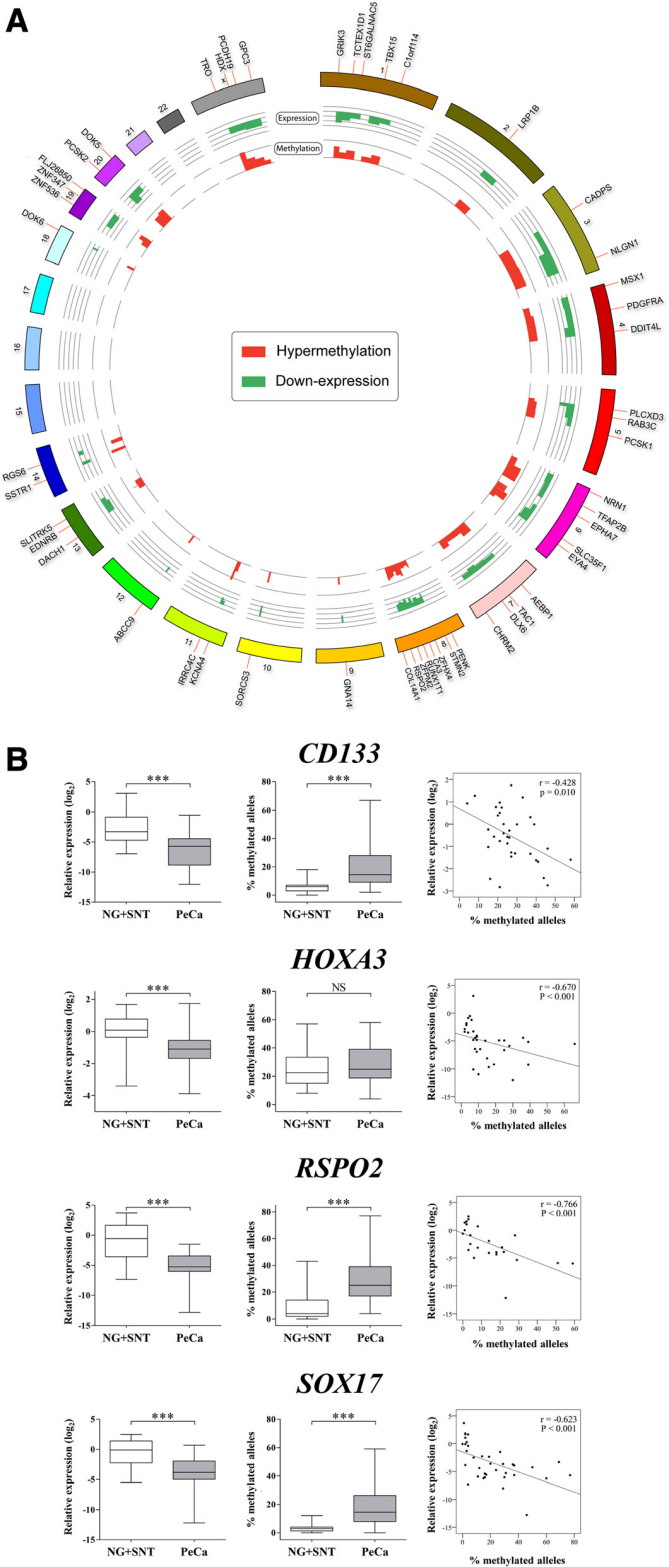
Circular plot and correlation graphics. **(A)** Circular representation of the genes with inverse correlation between gene expression and methylation. Fifty-four hypermethylated/underexpressed genes are shown. The tracks on the outside represent (1) genes, (2) chromosomes, (3) gene expression, and (4) methylation. **(B)** Graphic representation of gene expression, methylation, and Spearman correlation for the *SOX17*, *RSPO2*, *CD133*, and *HOXA3* genes. First column - relative gene expression levels by quantitative RT-PCR; second column - methylation levels by pyrosequencing; third column - Spearman correlation. NG: normal glans; SNT: surrounding non-malignant tissue; PeCa: penile carcinoma; NS: not significant; *: *P* value ≤0.05.

Integrative analysis was performed using both methylome and transcriptome data from 25 samples. This analysis revealed that 54 of the 106 hypermethylated genes (51%), including *RSPO2* and *SOX17,* also presented reduced levels of expression (Figure 2A), which was subsequently validated by pyrosequencing and RT-qPCR (Additional file 1: Figures S2 and S3).

Twenty probes (representing 20 genes) were selected for validation by pyrosequencing analysis. Paired (18 PeCa and SNT) and unpaired samples (44 PeCa, 30 SNT, and 11 NG) were included. Eighteen of 20 probes confirmed the MCIp-chip findings in the paired analysis, while all of them were validated in the unpaired samples (Additional file 1: Figure S2). Twelve of the 20 genes that were evaluated by pyrosequencing also underwent RT-qPCR analysis. Significant underexpression was observed for 8 genes (*TWIST1*, *RSPO2*, *SOX3*, *SOX17*, *PROM1*, *OTX2*, *HOXA3*, and *MEIS1)* (Additional file 1: Figure S3). Table 1 summarizes the results obtained from the microarray, pyrosequencing, and RT-qPCR analyses. Correlation analysis for methylation levels and gene expression detected by pyrosequencing and RT-qPCR showed a significant inverse correlation for the *SOX17*, *RSPO2*, *PROM1*, and *HOXA3* genes (Figure 2B)*.*

**Table 1:** **Differentially methylated genes as revealed by genome-wide methylation analysis, which were selected for validation by pyrosequencing and RT-qPCR**

**Genes**	**MCIp-chip**	**Pyrosequencing**	**Expression (RT-qPCR)**
***RSPO2***	•	•	Underexpressed
***TWIST1***	•	•	Underexpressed
SOX3*	•	•	Underexpressed
***SOX17***	•	•	Underexpressed
***PROM1/CD133***	•	•	Underexpressed
HOXA3*	•	•	Underexpressed
***MEIS1***	•	•	Underexpressed
***OTX2***	•	•	Underexpressed
***SOX14***	•	•	N.S.
***ONECUT1***	•	•	N.S.
***PAX7***	•	•	N.S.
***FOXA1***	•	•	N.S.
***LHX5***	•	•	-
***BDNF***	•	•	-
***NKX2.2***	•	•	-
***NKX2.3***	•	•	-
***CDX2***	•	•	-
***ONECUT2***	•	•	-
***LIN28A***	•	•	-
***WIT1***	•	•	-

•: methylated probe, *P* value ≤0.05; N.S.: not significant; − not performed; *For these genes, *P* value ≤0.05 was obtained from the Mann–Whitney test. For all other genes, significance was obtained using the Kruskal-Wallis test.

### Transcriptome and methylome profiles are influenced by HPV infection

Seventeen of the 44 PeCa (38.6%) evaluated in this study were HPV-positive, the vast majority for subtype 16 (88.2%). Unsupervised analysis revealed that HPV status influenced both gene expression levels and methylation patterns (Figure 1C, D). Three thousand forty-nine differentially methylated probes and 1,119 differentially expressed genes were identified in the HPV-positive cases when compared to HPV-negative cases (fold change > 1.5). Hierarchical clustering analysis of the transcriptome data revealed a cluster that comprised of solely HPV-positive PeCa (9/9 cases). The remaining HPV-positive cases clustered with HPV-negative samples (2/24). These clusters were noted to be significantly different when submitted to the chi-squared test (*P* value = 0.000005) (Figure 1C). Hierarchical clustering of the methylation data also revealed an HPV-positive tumor-enriched cluster (7/11 cases, 88%). Only one HPV-positive sample was observed to be grouped with the HPV-negative cases (1/14, 24%, *P* = 0.034) (Figure 1D). Integrative analysis performed on 25 cases (7 HPV-positive and 18 HPV-negative) revealed the presence of 65 inversely correlated genes (hypermethylated and underexpressed) in HPV-positive cases, including *CD70*, *HN1*, *FZD5*, *FSCN1*, and *PRR16* (correlation coefficient < −0.60 and *P* value < 0.05). Similarly, 60 genes were detected in the HPV-negative cases (Additional file 2: Table S3).

### Comparison between global methylation and expression profile and clinicopathological features

Unsupervised hierarchical clustering analysis of gene expression revealed a group of HPV-negative cases that were related to a poor prognosis, whether due to recurrence, lymph node metastasis, high histology grade, or death (Figure 1C). A distinct methylome profile was detected according to histological grading. Grade 2 and 3 tumors presented similar methylation profiles, which were different from grade 1 (Additional file 1: Figure S4A). The methylation profiles were particularly different when poorly differentiated (grade 3) were compared to well-differentiated tumors (grade 1) (Additional file 1: Figure S4B). This comparison revealed the presence of 122 differentially methylated probes in grade 3 tumors, including hypermethylated probes representing the *BCL3*, *OTOP1*, and *PAX2* genes. This methylome signature was considered unique to tumor grade, with none of the probes being altered in other comparisons.

Enrichment and upstream regulator analyses were performed for the 122 differentially methylated probes found for the grade 3 tumors. Extracellular matrix receptor interaction, cell migration, and developmental biology were the principal pathways identified (corrected *P* value <0.05, Additional file 2: Table S4).

The 20 genes selected for validation by pyrosequencing and the 12 genes selected for RT-qPCR were evaluated with regard to their clinical features. Univariate analysis revealed that low *NKX2-3* and *BDNF* methylation levels were associated to a shorter disease-free survival (Additional file 1: Figure S4C and D, respectively). Low *BDNF* methylation levels were also associated with lymph node metastases (*P* = 0.035). Low *LIN28A* (*P* = 0.023) and *CDX2* (*P* = 0.041) methylation levels were associated with advanced clinical stage (III and IV). In addition, *FOXA1* overexpression was associated with perineural invasion (*P* = 0.033), while *OTX2* overexpression was associated with angiolymphatic invasion (*P* = 0.040) (Additional file 2: Table S5). However, after multiple-comparison correction, these findings were not validated.

### *In silico* molecular enrichment analysis

Enrichment analysis, alongside the use of the Ingenuity Pathway Analysis (IPA) software, was used to identify gene function annotation and canonical pathways. These analyses were performed for the genes that presenting altered expression or methylation patterns, with the objective of gaining an insight into the onco-pathogenic pathways of PeCa. KOBAS and Gene Set Enrichment Analysis (GSEA) software were used to confirm the results (Additional file 2: Table S6). Hypermethylated genes were related to embryonic, cellular, and organism developmental functions. The most significant functions identified for the genes with altered expression were cell and embryonic development, cell movement, migration, growth and proliferation, cell cycle, and angiogenesis.

Pathway analysis, which was performed using IPA, KOBAS, and GSEA software, identified altered genes related to the molecular mechanisms of carcinoma, transcriptional regulatory networks in embryonic stem cells, Wnt/β-catenin signaling, and cell cycle. In addition, upstream regulator analysis of the differentially expressed genes was performed, revealing 40 altered genes, which were considered as involved in the activation/inactivation of 126 genes in PeCa (Additional file 2: Table S7). Among these genes, *PIK3CD*, *FGF1*, *ILIA*, *ILIB*, and *TNK* are already established therapeutic targets with the potential to be used in clinical practice.

## Discussion

Genome-wide studies revealed methylome signatures that can stratify cancer subtype [18], predict cancer outcome [19], and identify “driver” epigenetic events, which promote cancer cell survival [20]. In this study, genome-wide methylation analysis of penile cancer revealed 171 hypermethylated probes, 20 of which were validated by pyrosequencing. Hypomethylated probes were not identified, which may be partially explained by the experimental approach used that favored enrichment of methylated DNA. In addition, the microarray platform mainly focused on promoter regions and CpG islands and the filter used in the identification of the differentially methylated probes was very stringent. Furthermore, global hypomethylation of the PeCa genome was observed via the analysis of methylation levels of the AluYB8 and AIR1Sat repetitive sequences. This is consistent with the notion that global loss of methyl-cytosine is a universal cancer-associated phenomenon related to increased genomic instability [21].

Transcriptome analysis identified 4,261 differentially expressed genes. Six *MMP* genes (*MMP1*, *MMP7*, *MMP9*, *MMP10*, *MMP12*, and *MMP13*) were detected among the top 30 overexpressed transcripts (Additional file 2: Table S2). Accordingly, one of the functions identified by IPA, KOBAS, and GSEA was related to extracellular matrix dysregulation (Additional file 2: Table S6). In addition, the MMP-9 protein has been shown to be an independent risk factor for cancer recurrence in PeCa [22]. To explore the interactions between the transcripts, as well as to identify putative transcriptional regulators with the potential to be therapeutic targets, an upstream regulator analysis was performed. The prediction of this analysis was supported by the expected direction expression of the downstream factors in the input gene list. The differentially expressed transcripts predicted the inhibition of 17 genes and activation of 23 genes, which were shown to be under- and overexpressed, respectively in PeCa when compared to NG. The analysis revealed the *PIK3CD*, *FGF1*, *IL1A*, *IL1B*, and *TNF* genes as having the potential of being therapeutic targets (Additional file 2: Table S7).

Integrative analysis using methylome and transcriptome data revealed underexpression of 51% of the hypermethylated genes (54 of 106). In an independent group of samples, an inverse correlation was also observed between the methylation and transcript levels. The 20 probes selected for validation using pyrosequencing confirmed the results obtained in the microarray analysis. In addition, the expression levels for 8 of the 12 genes evaluated by RT-qPCR were inversely correlated with methylation status, suggesting that methylation is associated with dysregulation of these genes.

Although HPV infection is one of the risk factors associated to PeCa, few studies have evaluated its impact on DNA methylation changes. A recent study revealed that HPV-16 infection influenced the epigenetic profile of PeCa [17]. In the present study, 38.6% of the cases were HPV-positive. When comparing HPV-positive and negative PeCa, integrative analysis of the transcriptome and methylome revealed the presence of 65 hypermethylated and underexpressed genes in the HPV-positive tumors. In addition, unsupervised hierarchical clustering analysis revealed a specific group of genes in HPV-positive PeCa with altered methylation and expression levels, which is consistent with the hypothesis that HPV dysregulates specific signaling pathways that participate in penile carcinogenesis [8-10].

One of the interesting findings in this study was that aside HPV infection, other pathways, such as cellular and embryonic development and stem cell regulation could be involved in PeCa. Hypermethylation of several genes involved in cellular differentiation during embryonic development (*NKX2-2, NKX2-3*, *ONECUT1*, *ONECUT2*, *TWIST1*, *PROM1*, *MEIS1*, *HOXA3*, *CDX2*, *OTX2*, *FOXA1*, and *LHX5*) [23,24] was also identified. Accordingly, transcript levels for seven of these genes were evaluated, with five of them (*MEIS1*, *HOXA3*, *OTX2*, *TWIST1*, and *PROM1*) being underexpressed in the tumor samples. Aberrant methylation in several members of the SOX family genes, including *SOX1*, *SOX3*, *SOX9*, *SOX14*, *SOX17*, and *SOX20T*, was also observed. Recent evidence suggests that many *SOX* family genes physically interact with the β-catenin/Wnt signaling pathway, which regulates the transcription of target genes involved in embryonic patterning, development, and stem cell maintenance [25]. Furthermore, enrichment analysis revealed the transcriptional regulatory network in embryonic stem cells as the major canonical pathway involved in PeCa (*P* value = 0.00097).

Interestingly, the methylation profile of PeCa was capable of differentiating the samples according to histological grade. One hundred and twenty-two probes were differentially methylated in grade 3 tumors (Additional file 1: Figure S4), with the principal pathways involved in this group being extra cellular matrix receptor interaction and cell migration. Dysregulation of these pathways contribute to neoplastic progression and cell migration [26], supporting the results of the present study.

The strongest prognostic factor for PeCa is lymph node metastasis [4]. However, no useful clinical molecular markers to predict PeCa outcome are available. Prognostic markers for inguinal metastases could aid in the stratification of patients at higher risk. Thus, unnecessary prophylactic inguinal lymphadenectomy, which is associated with high morbidity, could be avoided. Univariate analysis revealed that low *BDNF* methylation was associated with lymph node metastasis and a shorter disease-free survival. Although further studies are required in order to validate this finding, we propose *BDNF* as a potential prognostic molecular marker for PeCa. Univariate analysis also identified other putative prognostic markers that could be used clinically (Additional file 2: Table S5). Multivariate analysis did not confirm these findings, probably due to the small sample size and number of adverse events (death/recurrence).

## Conclusions

This study identified novel epigenetically regulated genes in penile carcinogenesis. Several genes were validated and associated with transcript expression levels and clinical parameters. The dysregulated genes detected are known to be associated with essential cellular processes, which suggest that they play an important role in the development and progression of this disease. Our data also suggests an association between the involvement of the stem cell regulatory pathway to differential prognosis in PeCa. This study may prove instrumental in the discovery of biomarkers for clinical and molecular epidemiology of PeCa.

## Methods

### Subjects

Forty-four usual-type penile squamous cell carcinomas (SCC), surrounding non-malignant tissue (SNT; *N* = 30), and 13 normal glans (NG), the latter obtained from necropsy, from the AC Camargo Cancer Center, São Paulo, SP, Brazil, and Barretos Cancer Hospital, Barretos, SP, Brazil, were used. The SNT samples were obtained from as far away as possible from the tumor margin. They were composed mainly of squamous cells, considered histologically normal, and thus used as a reference in our experiments. Patient progress was monitored prospectively, with a mean follow-up of 20 months (1 to 67 months). Eighty-four percent of the patients were submitted to partial penectomy, with none having been treated prior to sample collection. Patients and/or family members were counseled regarding the procedure and subsequently provided written informed consent. The Human Research Ethics Committees of the aforementioned institutions approved the study. Clinical and histopathological data are summarized in Additional file 2: Table S8.

DNA from frozen penile tissue was isolated using the DNeasy Blood & Tissue Kit (Qiagen, Valencia, CA, USA), and RNA was extracted using RNeasy ™ (Qiagen). RNA integrity was confirmed using the Agilent 2100 Bioanalyzer RNA 6000 LabChip kit (Agilent Technologies, Santa Clara, CA, USA). HPV status was established for all PeCa via Linear Array HPV Test Genotyping (Roche Molecular Diagnostics, Branchburg, NJ, USA). Furthermore, primers binding to the L1 region of the HPV virus (primers pair GP5+/GP6+) were also used [27].

Twenty-five of the 44 PeCa samples and 10 SNT were evaluated using methylation microarray. Validation by quantitative bisulfite pyrosequencing was performed on samples used in the microarray (technical validation; 25 PeCa and 10 SNT), as well as an independent set of samples (biological validation; 19 PeCa, 20 SNT and 11 glans samples). Thirty-one PeCa RNA samples were used for gene expression profiling, while 39 PeCa were used for RT-qPCR analysis, of which, 8 PeCa, 13 SNT, and 8 glans were independent of global gene expression microarray. Detailed information of the sample selection is described in Additional file 1: Figure S1.

### Genome-wide methylation microarray

Enrichment of methylated sequences was performed for a subset of 25 PeCa and 10 SNT. The Methyl-CpG immunoprecipitation microarray (MCIp-chip) based protocol [28] was used to investigate methylated CpG-rich sequences (MethylMiner Methylated DNA Enrichment, Invitrogen Life Technologies, Carlsbad, CA, USA).

Genome-wide methylation assays were performed using the Agilent 244 K Human DNA Methylation Microarray (Agilent Technologies) as per the manufacturer’s recommendations. Workbench Standard (Ed. 5.0.14, Agilent Technologies) and BRB ArrayTools software (v. 4.2.1) [29] were used for microarray data normalization (Lowess) and statistical analyses. Genes whose methylation levels differed by at least 1.5 fold from the median in at least 20% of the arrays were retained. Using the random-variance *t*-test [30], differentially methylated genes were identified among the PeCa and SNT samples. The random-variance *t*-test is an improvement over the standard separate *t*-test and permits the sharing of information between genes concerning within-class variation without assuming that all genes have the same variance. Genes were considered statistically significant if their *P* value was less than 0.001. A stringent significance threshold was used to limit the number of false positive findings (FDR ≤0.05).

### Transcriptome analysis

Transcriptome analysis of 33 PeCa (labeled with Cy3) and a pool of five NG obtained from necropsies (labeled with Cy5) were evaluated using the Whole Human Genome 4 × 44K microarray platform (Agilent Technologies) according to the manufacturer’s recommendations. Images were acquired using a DNA microarray scanner (Agilent Technologies) and processed by the Feature Extraction Software (v. 10.1.1.1). Background-corrected mean signal intensity from each dye channel was used. Microarray data were normalized (Lowess) using the Agilent Feature Extraction Software (v.10.1.1.1). Additionally, a filter was applied to remove values with low reproducibility. Genes with a mean log_2_ signal ratio (Cy3/Cy5) of ≥ 1.0 and ≤ −1.0 within a 99% confidence interval (CI) were considered differentially expressed. Microarray data are available on the Gene Expression Omnibus (GEO) database (GSE57955). Integrative analysis was performed using the correlation function of the Hmisc package (http://cran.r-project.org).

### Quantitative bisulfite pyrosequencing and RT-qPCR

Validation using pyrosequencing included probes that were selected based on the following criteria: *P* value ≤ 0.001, FDR ≤ 0.05; more than one probe representing a gene promoter or CpG island; probe located in a CpG island; fold change > 1.5; and gene function related to cancer (based in Ingenuity Pathway Analysis (IPA) and Gene Set Enrichment Analysis (GSEA) software).

Assays were performed for 20 genes in 44 PeCa, 30 SNT, and 11 NG (Table 1). ALR1Sat and AluYB8 repetitive regions were assessed as previously described [31]. Bisulfite conversion of genomic DNA was performed (EZ DNA Methylation-Gold Kit, Zymo Research Corporation, Irvine, CA, USA). Regions flanking the altered probes were amplified using PCR (HotStarTaq Master Mix kit - Qiagen) and sequenced by pyrosequencing as per the manufacturer’s instructions (PyroMark ID Q96, Qiagen and Biotage, Uppsala, Sweden). For each gene selected, an average of four CpG nucleotides were investigated involving the probe represented in the microarray (Additional file 2: Table S9). Within each pyrosequencing assay, bisulfite conversion controls in the dispensation order sequence were included. To assure efficiency, 100% and 0% methylated DNA were also included in each run (Zymo Research Corporation).

A total of 39 PeCa, 13 SNT, and 13 NG were used for RT-qPCR. cDNA synthesis and amplification were performed as previously described [32]. Twelve of 20 genes investigated by pyrosequencing were selected for RT-qPCR validation (Table 1). All genes presented a fold change > 1.5 in the microarray results. In order to normalize target gene expression, the *HMBS* and *GUSB* genes were selected by the geNorm software [33] from six reference candidates (*ACTB*, *GAPDH*, *GUSB*, *HMBS*, *HPRT*, and *RPLP0*) (Additional file 2: Table S10). Relative quantification of gene expression was calculated according to Pfaffl [34].

### Statistical analysis

Fisher’s exact test was applied to compare categorical variables, while non-parametric paired (Wilcoxon signed-rank test), unpaired (Mann–Whitney *U*-test), multiple-comparison (Kruskal-Wallis with Post Hoc Dunn’s test), and correlation (Spearman) tests were used to compare continual data (RT-qPCR and pyrosequencing results) with clinical-pathological variables. In order to adjust the *P* value for the number of hypotheses tested, Bonferroni’s correction was applied. The Kaplan-Meier method alongside the log-rank test was used to compare overall cancer-specific and disease-free survival between groups. Multivariate analysis was performed with Cox proportional hazards, including variables with *P* value <0.2 in the univariate analysis. Significance was considered as two-tailed *P* value <0.05. Data from the paired and unpaired tests were analyzed using SPSS 17.0 (SPSS Inc; Chicago, IL, USA) and Graphpad Prism 5.0 (GraphPad Software Inc., La Jolla, CA, USA).

The hierarchical clustering analysis (HCL) method was used for the clustering analysis. Each cluster obtained was associated to the clinical data, with the comparison between clusters being performed by non-parametric chi-square (*χ*^2^) test.

### *In silico* molecular interaction analysis

The biological function, canonical pathways, and upstream regulators were investigated by an *in silico* molecular interaction analysis using Ingenuity Pathways Analysis (IPA, Ingenuity® Systems) software. Biological function and canonical pathways were confirmed via Gene Set Enrichment Analysis (GSEA) and KEGG Orthology Based Annotation System (KOBAS), which use the BioCyc, KEGG Pathway, Pathway Interaction Database, Reactome, and Panther databases. An FDR < 0.05 and corrected *P* value < 0.05 were considered for GSEA and KOBAS, respectively.

## Additional files

Additional file 1: Figure S1 to S4. **Figure S1.** Flow chart of experimental design. Eighty-seven samples were obtained: 44 PeCa, 30 SNT, and 13 glans. Of the 44 PeCa samples, 25 were used for methylation screening and 33 RNA samples for gene expression profiling. Several genes were selected for further evaluation by RT-qPCR (12 genes) and pyrosequencing (20 genes) in a microarray-dependent and independent set of samples. PeCa: Penile carcinoma; SNT: surrounding non-malignant tissue; NG: normal glans. **Figure S2.** Dot plot of pyrosequencing analysis. Graphics show the methylation levels of the ALR1Sat and AluYB8 regions and 20 probes (representing 20 genes) in NG, SNT, and PeCa samples (unpaired analysis). NG: normal glans tissue; SNT: surrounding non-malignant tissue; PeCa: penile carcinoma; ns: not significant; *: *P* value ≤0.05. Figure S3. Relative expression of 12 selected genes by RT-qPCR. NG: normal glans tissues; SNT: surrounding non-malignant tissue; PeCa: penile carcinoma; ns: not significant; *: *P* value ≤ 0.05. Mann Whitney test was applied for *SOX3* (*P* value ≤0.05) to compare SNT vs PeCa. For all other genes, the Kruskal-Wallis test was applied. Figure S4. Methylation heat maps according to tumor grade (1, 2, and 3) and Kaplan-Meier curves in relation to *NKX2-3* and *BDNF* methylation levels. Comparison between tumor grades revealed significant differences in methylation levels. A) Difference between histological tumor grades 1, 2, and 3. B) Grade 1 tumors revealed a distinct methylation profile when compared to grade 3 tumors. C and D) Low methylation levels of the *NKX2-3* and *BDNF* genes were associated with a shorter disease-free survival. Survival analysis was performed with methylation categorized for each gene as: high level, one standard deviation above the mean of the non-tumor group; or low level, one standard deviation below the mean of the non-tumor group. Additional file 2: Table S1 to S10. **Table S1.** List of hypermethylated probes. ID for NCBI access, probe ID of the altered probe, FDR, fold change, and chromosome position. Table S2. Top 100 differentially expressed genes (under- and overexpressed) in PeCa. Table S3. Integrative analysis for transcriptome data and methylation profile, according to HPV status. Table S3. Integrative analysis for transcriptome data and methylation profile, according to HPV status. Table S4. Top ranked pathways obtained from the 122 differentially methylated probes for the grade 3 tumors. KOBAS and GSEA software were used for the enrichment analysis. Table S5. Comparison between gene methylation pattern and transcript levels with clinical and pathologic parameters. Table S6. Top ranked functions and canonical pathways as identified by *in silico* analysis. Under- and overexpressed and hypermethylated genes were used as input in the IPA, KOBAS, and GSEA software. Table S7. Genes provided from the upstream regulator analysis (Ingenuity Pathways Analysis software (IPA)). Genes in the first column were altered (under- or overexpressed) in PeCa and are known to regulate several genes identified as differentially expressed in the transcriptome analysis. Table S8. Clinical and pathological characteristics of the penile carcinomas patients. Table S9. Primer sequences and probe properties for those evaluated by pyrosequencing. Table S10. Primer sequences and transcript properties for those evaluated by RT-qPCR.

## Abbreviations

CI: Confidence interval
FDR: False discovery rate
HCL: Hierarchical clustering analysis
IPA: Ingenuity pathway analysis
GSEA: Gene set enrichment analysis
KOBAS: KEGG orthology based annotation system
MCIp: Methyl-CpG immunoprecipitation
MMPs: Matrix metalloproteinases
NG: Normal glans
PeCa: Penile carcinoma
SNT: Surrounding non-malignant tissues
HPV: Human papillomavirus
