# Supplementary material for: Genome-wide methylation and transcriptome analysis in penile carcinoma: uncovering new molecular markers
Source: Clin Epigenetics. 2015 Apr 18;7(1):46. doi: 10.1186/s13148-015-0082-4 (PMC4407795; doi:10.1186/s13148-015-0082-4)
Supplement: Additional file 1: — Figure S1 to S4. Figure S1. Flow chart of experimental design. Eighty-seven samples were obtained: 44 PeCa, 30 SNT, and 13 glans. Of the 44 PeCa samples, 25 were used for methylation screening and 33 RNA samples for gene expression profiling. Several genes were selected for further evaluation by RT-qPCR (12 genes) and pyrosequencing (20 genes) in a microarray-dependent and independent set of samples. PeCa: Penile carcinoma; SNT: surrounding non-malignant tissue; NG: normal glans. Figure S2. Dot plot of pyrosequencing analysis. Graphics show the methylation levels of the ALR1Sat and AluYB8 regions and 20 probes (representing 20 genes) in NG, SNT, and PeCa samples (unpaired analysis). NG: normal glans tissue; SNT: surrounding non-malignant tissue; PeCa: penile carcinoma; ns: not significant; *: P value ≤0.05. Figure S3. Relative expression of 12 selected genes by RT-qPCR. NG: normal glans tissues; SNT: surrounding non-malignant tissue; PeCa: penile carcinoma; ns: not significant; *: P value ≤ 0.05. Mann Whitney test was applied for SOX3 (P value ≤0.05) to compare SNT vs PeCa. For all other genes, the Kruskal-Wallis test was applied. Figure S4. Methylation heat maps according to tumor grade (1, 2, and 3) and Kaplan-Meier curves in relation to NKX2-3 and BDNF methylation levels. Comparison between tumor grades revealed significant differences in methylation levels. A) Difference between histological tumor grades 1, 2, and 3. B) Grade 1 tumors revealed a distinct methylation profile when compared to grade 3 tumors. C and D) Low methylation levels of the NKX2-3 and BDNF genes were associated with a shorter disease-free survival. Survival analysis was performed with methylation categorized for each gene as: high level, one standard deviation above the mean of the non-tumor group; or low level, one standard deviation below the mean of the non-tumor group. [file 13148_2015_82_MOESM1_ESM.pdf]

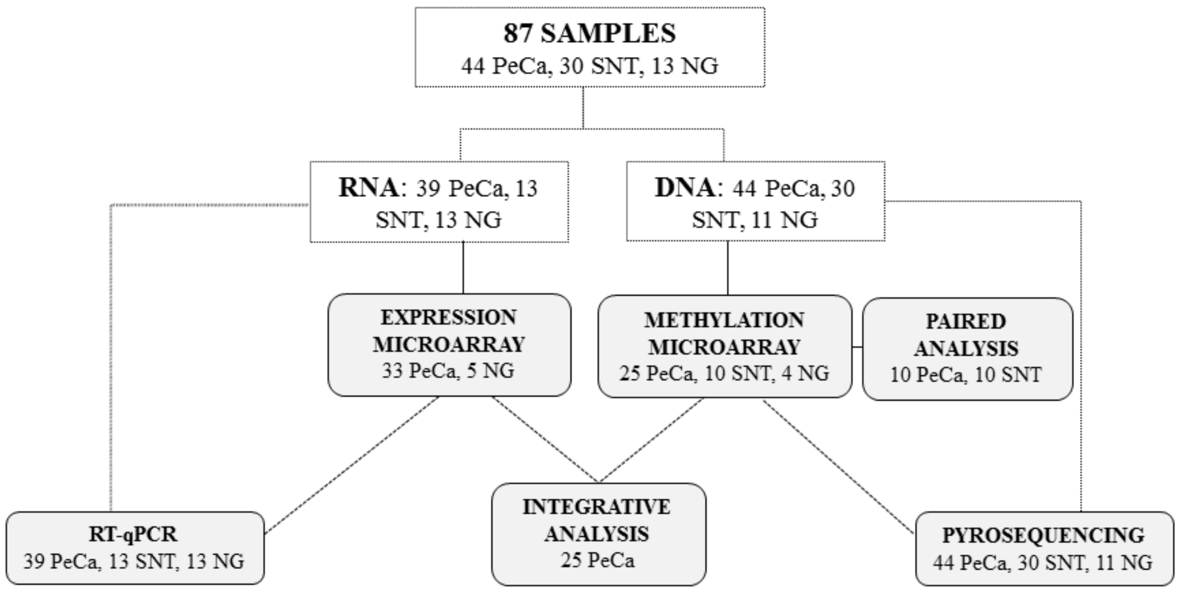

Figure S1. Flow chart of experimental design. Seventy-seven samples were obtained: 44 PeCa, 30 SNT and 13 glans. Of the 44 PeCa samples, 25 were used for methylation screening and 33 RNA samples for gene expression profiling. Several genes were selected for further evaluation by RT-qPCR (12 genes) and pyrosequencing (20 genes) in a microarray-dependent and independent set of samples. PeCa: Penile carcinoma; SNT: surrounding non-malignant tissue; NG: normal glans.

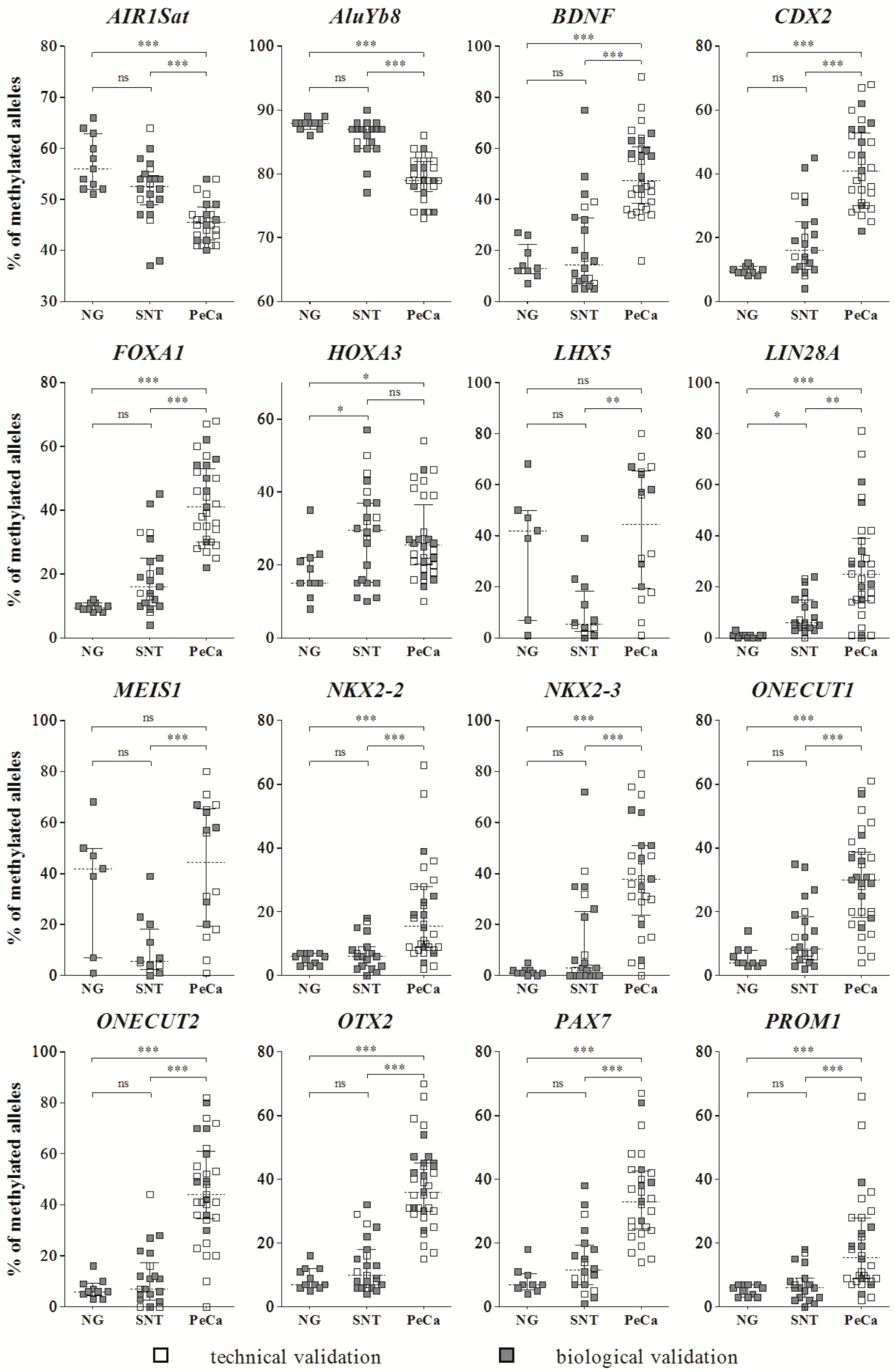

AIR1Sat
AluYb8
BDNF
CDX2
FOXA1
HOXA3
LHX5
LIN28A
MEIS1
NKX2-2
NKX2-3
ONECUT1
ONECUT2
OTX2
PAX7
PROM1
% of methylated alleles
NG
SNT
PeCa
technical validation
biological validation

Cont.

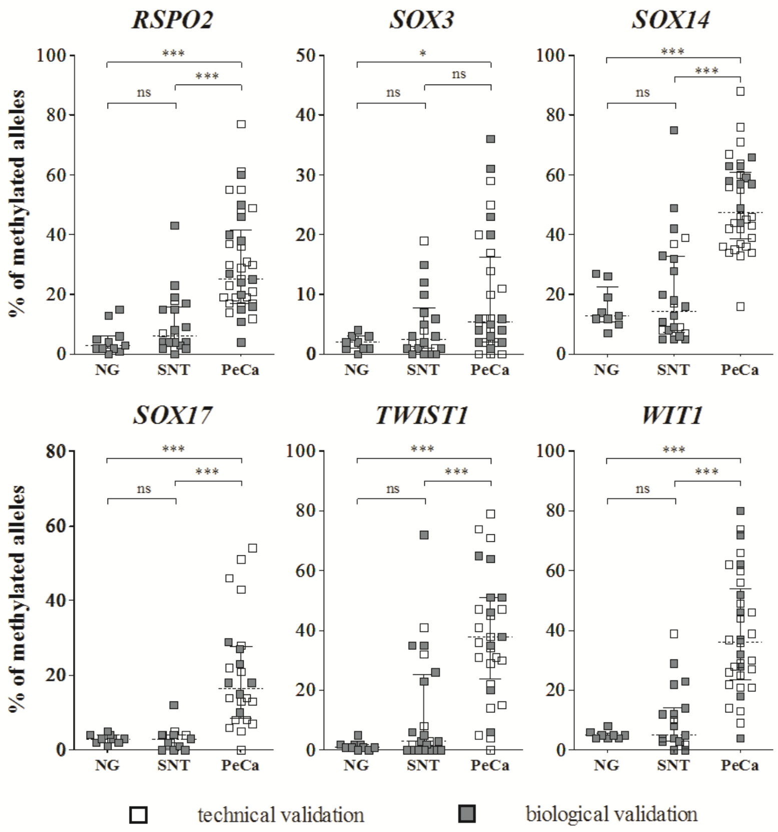

Figure S2. Dot Plot of pyrosequencing analysis. Graphics show the methylation levels of the ALR1Sat and AluYB8 regions and 20 probes (representing 20 genes) in NG, SNT and PeCa samples (unpaired analysis) NG: normal glans tissue; SNT: surrounding non-malignant tissue; PeCa: penile carcinoma; ns: not significant; *: p value ≤ 0.05.

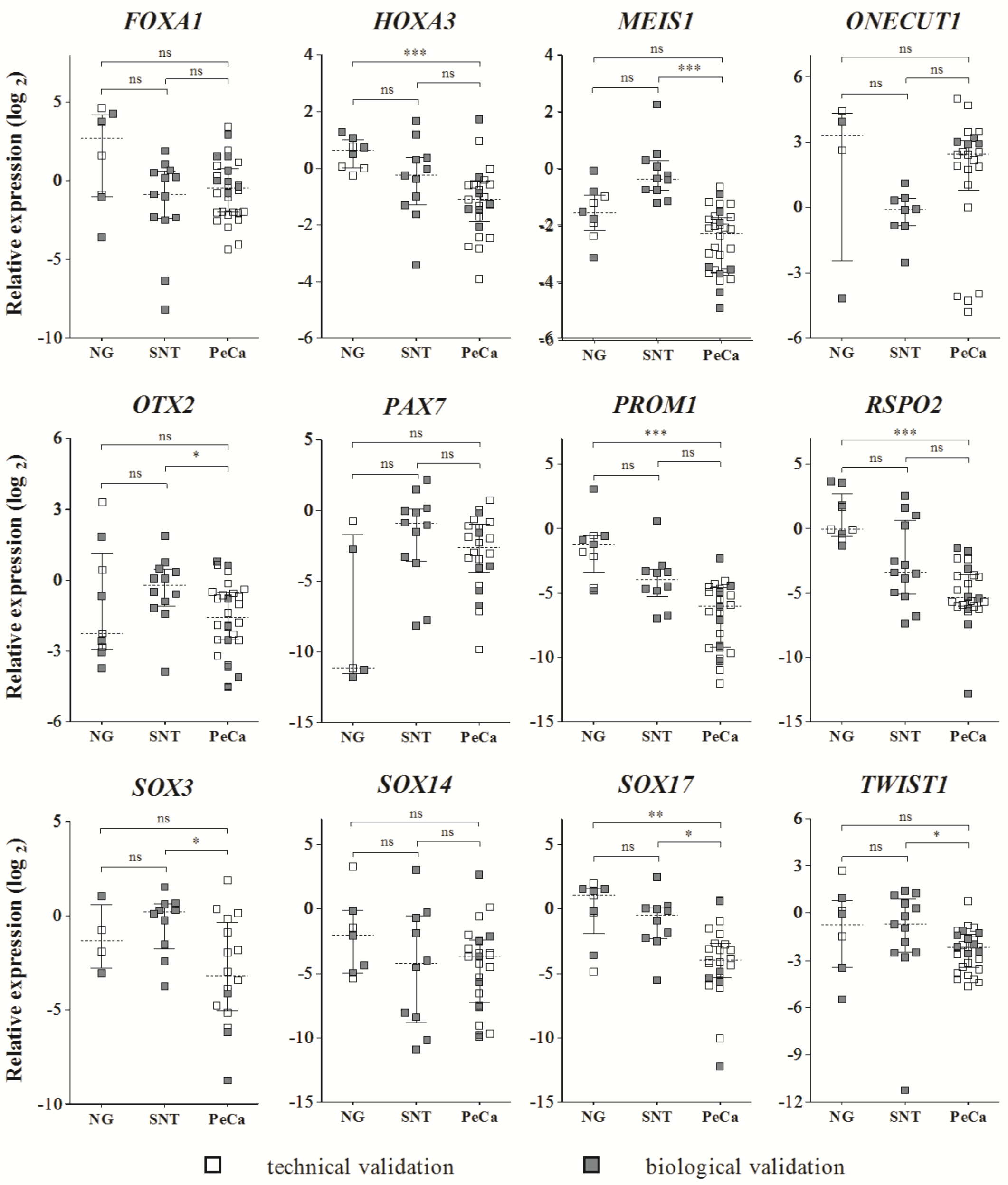

Figure S3. Relative expression of 12 selected genes by RT-qPCR. NG: normal glans tissues; SNT: surrounding non-malignant tissue; PeCa: penile carcinoma; ns: not significant; *: p value $\leq$ 0.05. Mann Whitney test was applied for *SOX3* (p value $\leq$ 0.05) to compare SNT vs PeCa. For all other genes the Kruskall-Wallis test was applied.

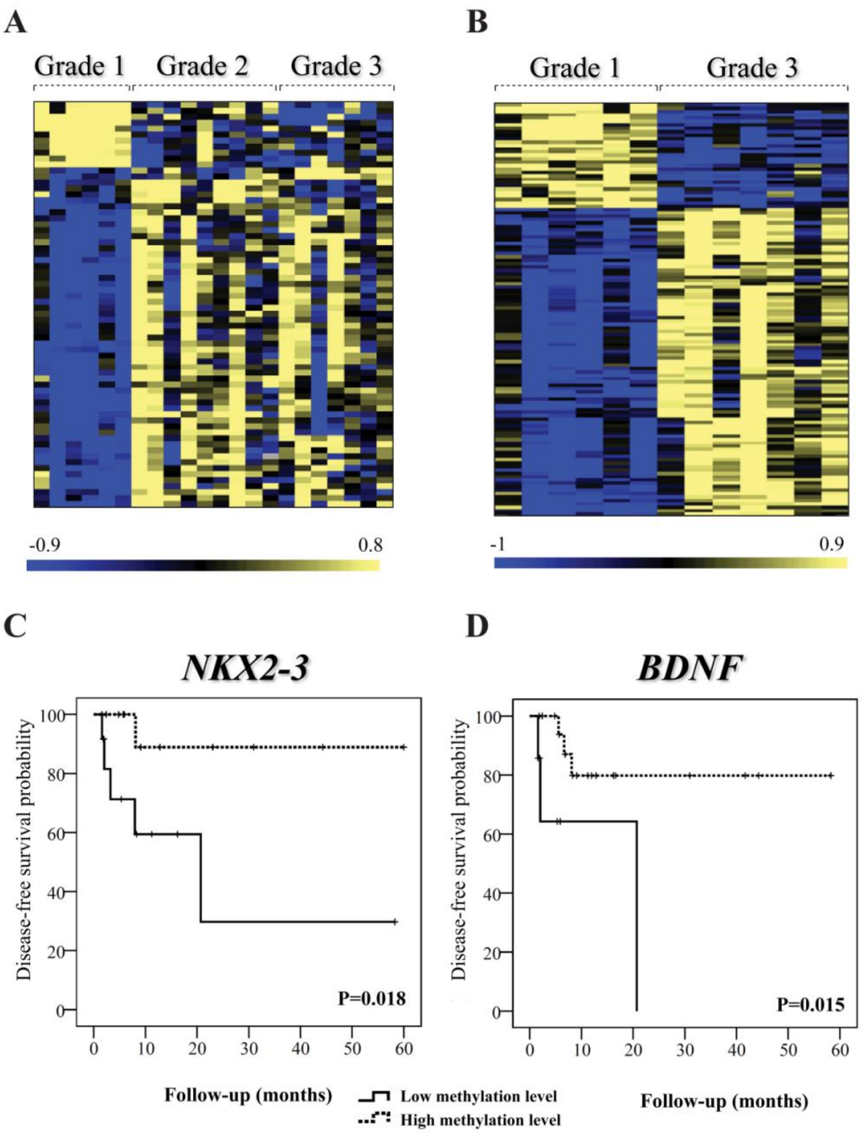

Figure S4. Methylation heat maps according to tumor grade (1, 2 and 3) and Kaplan-Meier curves in relation to *NKX2-3* and *BDNF* methylation levels. Comparison between tumor grades revealed significant differences in methylation levels. **A)** Difference between histological tumor grades 1, 2 and 3. **B)** Grade 1 tumors revealed a distinct methylation profile when compared to grade 3 tumors. **C and D)** Low methylation levels of the *NKX2-3* and *BDNF* genes were associated with a shorter disease-free survival. Survival analysis was performed with methylation categorized for each gene as: high level, one standard deviation above the mean of the non-tumor group; or low level, one standard deviation below the mean of the non-tumor group.
