# Supplementary material for: Genome-wide methylation and transcriptome analysis in penile carcinoma: uncovering new molecular markers
Source: Clin Epigenetics. 2015 Apr 18;7(1):46. doi: 10.1186/s13148-015-0082-4 (PMC4407795; doi:10.1186/s13148-015-0082-4)
Supplement: Additional file 2: — Table S1 to S10. Table S1. List of hypermethylated probes. ID for NCBI access, probe ID of the altered probe, FDR, fold change, and chromosome position. Table S2. Top 100 differentially expressed genes (under- and overexpressed) in PeCa. Table S3. Integrative analysis for transcriptome data and methylation profile, according to HPV status. Table S3. Integrative analysis for transcriptome data and methylation profile, according to HPV status. Table S4. Top ranked pathways obtained from the 122 differentially methylated probes for the grade 3 tumors. KOBAS and GSEA software were used for the enrichment analysis. Table S5. Comparison between gene methylation pattern and transcript levels with clinical and pathologic parameters. Table S6. Top ranked functions and canonical pathways as identified by in silico analysis. Under- and overexpressed and hypermethylated genes were used as input in the IPA, KOBAS, and GSEA software. Table S7. Genes provided from the upstream regulator analysis (Ingenuity Pathways Analysis software (IPA)). Genes in the first column were altered (under- or overexpressed) in PeCa and are known to regulate several genes identified as differentially expressed in the transcriptome analysis. Table S8. Clinical and pathological characteristics of the penile carcinomas patients. Table S9. Primer sequences and probe properties for those evaluated by pyrosequencing. Table S10. Primer sequences and transcript properties for those evaluated by RT-qPCR. [file 13148_2015_82_MOESM2_ESM.pdf]

Table S1. List of hypermethylated probes. ID for NCBI access, probe ID of the altered probe, FDR, fold-change and chromosome position.

Symbol	*Gene ID*	*ProbeID*	*FDR*	*Fold Change*	*MapLocation*	*Gene function*
ABCC9	10060	A_17_P08238636	0.036	2.3	chr12:21985847-21985891	Ion channel
ACTA1	58	A_17_P15190055	0.036	2.0	chr1:227636359-227636403	Ion channel
ADCY8	114	A_17_P26758093	0.036	2.6	chr8:132122607-132122652	Enzyme
		A_17_P06475866	0.045	2.1	chr8:132121709-132121753	
		A_17_P06475878	0.045	2.4	chr8:132123367-132123411	
ATP4A	495	A_17_P10948156	0.05	1.9	chr19:40740788-40740833	Transporter
BARHL1	56751	A_17_P27338364	0.039	2.1	chr9:134446538-134446582	Homeodomin protein/transcript factor
BARHL2	343472	A_17_P20400537	0.046	1.8	chr1:90954618-90954662	Homeodomin protein/transcript factor
BARX1	56033	A_17_P16242168	0.044	2.1	chr9:95750945-95750989	Homeodomin protein/transcript factor
BDNF	627	A_17_P07712649	0.039	2.0	chr11:27700398-27700442	Citokine and growth factor
		A_17_P07712647	0.039	2.0	chr11:27700255-27700309	
C14orf39	317761	A_17_P16707753	0.049	1.9	chr14:60022277-60022321	Other
C1orf114	57821	A_17_P00585874	0.036	2.0	chr1:167663430-167663482	Other
		A_17_P00585873	0.047	2.0	chr1:167663319-167663366	
C20orf56	140828	A_17_P11115750	0.036	2.3	chr20:22505444-22505502	Other
		A_17_P31759553	0.041	1.9	chr20:22507830-22507876	
CACNG3	10368	A_17_P16869900	0.036	2.0	chr16:24174578-24174622	Ion channel
CACNG7	59284	A_17_P10998631	0.038	2.1	chr19:59137279-59137323	Ion channel
CACNG8	59283	A_17_P10998740	0.036	2.2	chr19:59175408-59175452	Ion channel
CALCA	796	A_17_P07656161	0.048	1.9	chr11:14951845-14951889	Citokine and growth factor
CDX2	1045	A_17_P08741209	0.044	1.9	chr13:27439498-27439557	Homeodomin protein/transcript factor
CNTNAP5	129684	A_17_P21569681	0.038	2.4	chr2:124498973-124499023	Other
		A_17_P15318263	0.047	1.9	chr2:124499051-124499107	
COL14A1	7373	A_17_P06427847	0.038	2.0	chr8:121206780-121206825	Other
		A_17_P16130592	0.045	2.1	chr8:121206852-121206896	
CRISPLD1	83690	A_17_P06221585	0.036	2.2	chr8:76059263-76059307	Other

Cont.

Symbol	*Gene ID*	*UniqueID*	*FDR*	*Fold change*	*MapLocation*	*Gene function*
CSMD3	114788	A_17_P06395993	0.036	2.2	chr8:114514964-114515008	Enzyme
		A_17_P06395991	0.043	1.8	chr8:114514801-114514845	
		A_17_P26678353	0.049	2.1	chr8:114518098-114518157	
DBX1	120237	A_17_P07680059	0.036	2.3	chr11:20138642-20138698	Homeodomin protein/transcript factor
		A_17_P07680061	0.047	1.9	chr11:20138810-20138854	
DGKG	1608	A_17_P15520017	0.047	1.9	chr3:187561933-187561977	Protein kinase
DKFZP434H168	26077	A_17_P16899074	0.044	2.1	chr16:54785433-54785485	Other
DLX6	1750	A_17_P15991490	0.046	2.0	chr7:96465142-96465186	Homeodomin protein/transcript factor
DOK5	55816	A_17_P11222263	0.036	2.2	chr20:52526358-52526402	Other
EN2	2020	A_17_P05887712	0.04	2.0	chr7:154937070-154937114	Homeodomin protein/transcript factor
EPHA7	2045	A_17_P04903793	0.049	2.1	chr6:94184190-94184234	Protein kinase
EVX2	344191	A_17_P01695014	0.039	2.0	chr2:176658067-176658119	Homeodomin protein/transcript factor
		A_17_P01695013	0.045	2.0	chr2:176657948-176658006	
FAM150A	389658	A_17_P16094843	0.048	1.9	chr8:53640046-53640097	Other
FOXB2	442425	A_17_P27091196	0.047	2.1	chr9:78826240-78826295	Transcript factor
FOXC2	2303	A_17_P10170658	0.047	2.0	chr16:85157228-85157274	Transcript factor
GAD1	2571	A_17_P21794121	0.036	2.3	chr2:171378696-171378743	Enzyme
		A_17_P21794195	0.045	2.0	chr2:171387447-171387498	
GDF6	392255	A_17_P06316227	0.044	2.1	chr8:97226850-97226903	Citokine and growth factor
		A_17_P06316316	0.047	2.0	chr8:97238934-97238978	
		A_17_P06316318	0.047	2.2	chr8:97239280-97239333	
GPR139	124274	A_17_P09952877	0.05	1.8	chr16:19993075-19993123	G-protein coupled receptor
HCRTR2	3062	A_17_P04752213	0.047	2.0	chr6:55147181-55147228	G-protein coupled receptor
HDX	139324	A_17_P11808937	0.049	1.8	chrX:83643928-83643972	Other
HMX2	3167	A_17_P07539294	0.045	1.9	chr10:124893115-124893159	Homeodomin protein/transcript factor
KCNA4	3739	A_17_P28123519	0.036	2.1	chr11:29994227-29994279	Ion channel
		A_17_P07722804	0.036	2.2	chr11:29994350-29994401	

Cont.

Symbol	*Gene ID*	*UniqueID*	*FDR*	*Fold change*	*MapLocation*	*Gene function*
KCNV1	27012	A_17_P06380055	0.047	1.9	chr8:111056151-111056195	Ion channel
LHX2	9355	A_17_P06954369	0.045	2.0	chr9:125818993-125819038	Homeodomin protein/transcript factor
LHX5	64211	A_17_P29058043	0.036	2.1	chr12:112386029-112386073	Homeodomin protein/transcript factor
LHX8	431707	A_17_P00299472	0.047	1.8	chr1:75368569-75368615	Homeodomin protein/transcript factor
LRAT	9227	A_17_P03544555	0.036	2.0	chr4:155883760-155883804	Enzyme
LRP1B	53353	A_17_P01528538	0.047	1.8	chr2:142604347-142604400	Transmembrane receptor
LRRC4C	57689	A_17_P28170279	0.036	2.4	chr11:40271359-40271405	Other
MEOX2	4223	A_17_P05323186	0.044	2.0	chr7:15693422-15693466	Homeodomin protein/transcript factor
MGC45800	90768	A_17_P15644051	0.047	2.6	chr4:183300448-183300492	Other
MOS	4342	A_17_P06135989	0.047	2.3	chr8:57188271-57188323	Protein kinase
MSX1	4487	A_17_P02902305	0.046	2.0	chr4:4910130-4910178	Homeodomin protein/transcript factor
NID2	22795	A_17_P29750410	0.038	2.0	chr14:51604752-51604796	Other
NKX2-2	4821	A_17_P11110586	0.036	2.3	chr20:21438392-21438439	Homeodomin protein/transcript factor
		A_17_P11110666	0.036	2.1	chr20:21449874-21449918	
		A_17_P11110672	0.037	2.2	chr20:21451830-21451882	
		A_17_P17179057	0.039	2.1	chr20:21449497-21449541	
		A_17_P11110620	0.04	1.9	chr20:21443698-21443742	
		A_17_P11110664	0.044	2.1	chr20:21449392-21449451	
NKX3-2	579	A_17_P02936292	0.045	2.0	chr4:13150778-13150825	Homeodomin protein/transcript factor
NLGN1	22871	A_17_P02775610	0.036	2.3	chr3:174785418-174785467	Enzyme
		A_17_P02775615	0.045	2.0	chr3:174785829-174785875	
NOVA1	4857	A_17_P09182545	0.036	2.2	chr14:26137697-26137744	Other
NPHS1	4868	A_17_P17134237	0.045	1.9	chr19:41027056-41027100	Other
NRK	203447	A_17_P11866457	0.047	1.8	chrX:104953217-104953261	Protein kinase
NXPH1	30010	A_17_P25505781	0.036	2.1	chr7:8450275-8450327	Other
		A_17_P05288557	0.036	2.0	chr7:8449923-8449967	
OLIG2	10215	A_17_P17231917	0.047	2.0	chr21:33319865-33319909	Transcript factor

Cont.

Symbol	*Gene ID*	*UniqueID*	*FDR*	*Fold change*	*MapLocation*	*Gene function*
ONECUT1	3175	A_17_P09659699	0.049	1.8	chr15:50874085-50874129	Homeodomin protein/transcript factor
ONECUT2	9480	A_17_P10736367	0.044	2.0	chr18:53257093-53257144	Homeodomin protein/transcript factor
OTX2	5015	A_17_P29772123	0.036	2.1	chr14:56348893-56348940	Homeodomin protein/transcript factor
PARP15	165631	A_17_P02548080	0.05	2.2	chr3:123779262-123779306	Other
PAX7	5081	A_17_P15033933	0.036	2.1	chr1:18832427-18832476	Homeodomin protein/transcript factor
PCDH17	27253	A_17_P08876699	0.036	2.3	chr13:57103840-57103889	Other
		A_17_P08876698	0.041	2.0	chr13:57103674-57103726	
PCSK1	5122	A_17_P04124108	0.045	2.0	chr5:95794765-95794816	Peptidase
PDE4B	5142	A_17_P00258089	0.05	1.9	chr1:66031654-66031698	Enzyme
PDGFRA	5156	A_17_P23258860	0.044	1.9	chr4:54793027-54793082	Citokine and growth factor
PRDM14	63978	A_17_P26482035	0.043	2.1	chr8:71145424-71145468	Transcript factor
PRHOXNB	646625	A_17_P08741289	0.045	2.1	chr13:27450864-27450908	Enzyme
PUS3	83480	A_17_P08097861	0.036	2.1	chr11:125278869-125278924	Enzyme
RAB3C	115827	A_17_P03953335	0.045	2.1	chr5:57915223-57915275	Enzyme
		A_17_P03953334	0.047	2.0	chr5:57915151-57915201	
RASGRF1	5923	A_17_P09777987	0.038	2.1	chr15:77169713-77169757	Other
RFX4	5992	A_17_P08596300	0.041	1.9	chr12:105502893-105502940	Transcript factor
RGS20	8601	A_17_P06126588	0.045	2.0	chr8:54952819-54952870	Other
RGS6	9628	A_17_P09380532	0.044	2.0	chr14:71469728-71469776	Enzyme
		A_17_P29840048	0.049	2.0	chr14:71469890-71469934	
RORB	6096	A_17_P06741116	0.039	2.2	chr9:76304689-76304738	Transcript factor
		A_17_P06741115	0.044	1.9	chr9:76304615-76304663	
RSPO2	340419	A_17_P26653858	0.036	2.5	chr8:109165110-109165154	Receptor Binding
		A_17_P06371131	0.036	2.2	chr8:109164379-109164429	
		A_17_P26653843	0.041	2.1	chr8:109163335-109163388	
SCG3	29106	A_17_P30147107	0.036	2.1	chr15:49761061-49761105	Other

Cont.

Symbol	*Gene ID*	*UniqueID*	*FDR*	*Fold change*	*MapLocation*	*Gene function*
		A_17_P09227023	0.036	2.3	chr14:36053145-36053189	
SFTA3	253970	A_17_P09227024	0.036	2.2	chr14:36053271-36053320	Other
		A_17_P09227026	0.044	2.0	chr14:36053489-36053543	
SLC24A2	25769	A_17_P09227026	0.044	2.0	chr14:36053489-36053543	Transporter
SLC32A1	140679	A_17_P11157911	0.036	2.0	chr20:36788766-36788811	
		A_17_P11157908	0.036	2.0	chr20:36788577-36788624	Transporter
		A_17_P11157930	0.043	2.0	chr20:36791055-36791101	
		A_17_P11157916	0.044	1.9	chr20:36789240-36789284	
SLITRK3	22865	A_17_P02736025	0.036	2.2	chr3:166396056-166396100	
		A_17_P22876066	0.044	2.0	chr3:166395829-166395874	Other
		A_17_P22876069	0.049	2.0	chr3:166396212-166396266	
		A_17_P09018898	0.036	2.2	chr13:87123799-87123852	
SLITRK5	26050	A_17_P09018901	0.036	2.0	chr13:87124098-87124154	Other
		A_17_P09018899	0.049	1.9	chr13:87123916-87123964	
SNTG1	54212	A_17_P16093546	0.047	2.2	chr8:50985855-50985908	Other
SOX14	8403	A_17_P22753722	0.034	2.4	chr3:138963224-138963270	Transcript factor
SOX17	64321	A_17_P26412159	0.041	2.0	chr8:55528683-55528729	Transcript factor
SOX2OT	347689	A_17_P02813547	0.038	2.1	chr3:182928302-182928361	Transcript factor
SOX3	6658	A_17_P11991249	0.044	1.8	chrX:139416332-139416378	Transcript factor
SSTR1	6751	A_17_P09235148	0.045	2.0	chr14:37747511-37747555	G-protein coupled receptor
ST6GALNAC5	81849	A_17_P00307347	0.049	1.9	chr1:77105597-77105656	Enzyme
STMN2	11075	A_17_P06243029	0.037	2.1	chr8:80686506-80686553	Other
TBX15	6913	A_17_P00489877	0.039	2.1	chr1:119331588-119331632	Transcript factor
TMEM196	256130	A_17_P05343711	0.036	2.3	chr7:19779448-19779492	Other
TP53TG5	27296	A_17_P11187276	0.049	2.0	chr20:43435925-43435984	Other
TWIST1	7291	A_17_P05340624	0.034	2.3	chr7:19113600-19113655	
		A_17_P05340626	0.036	2.4	chr7:19113800-19113844	Transcript factor
		A_17_P05340625	0.036	2.1	chr7:19113678-19113723	

Cont.

Symbol	*Gene ID*	*UniqueID*	*FDR*	*Fold change*	*MapLocation*	*Gene function*
WIT1	51352	A_17_P07734872	0.036	2.3	chr11:32414288-32414339	Other
		A_17_P07734871	0.036	2.0	chr11:32414223-32414268	
		A_17_P07734875	0.044	2.0	chr11:32414631-32414675	
		A_17_P07734876	0.044	2.0	chr11:32414741-32414789	
		A_17_P07734806	0.047	2.0	chr11:32405844-32405903	
ZFPM2	23414	A_17_P06356935	0.039	2.1	chr8:106399422-106399473	Transcript factor
ZIC1	7545	A_17_P02656334	0.047	1.9	chr3:148612404-148612452	Transcript factor
ZIC4	84107	A_17_P02656172	0.047	1.9	chr3:148593204-148593248	Transcript factor
ZIC5	85416	A_17_P09077784	0.036	2.0	chr13:99422842-99422887	Transcript factor
ZNF347	84671	A_17_P31637802	0.039	1.9	chr19:58354104-58354148	Other
ZNF415	55786	A_17_P10996998	0.047	1.8	chr19:58327872-58327916	Transcript factor
		A_17_P31637698	0.047	1.9	chr19:58327806-58327850	
ZNF578	147660	A_17_P10996139	0.047	2.1	chr19:57648767-57648822	Other
		A_17_P10996138	0.05	1.9	chr19:57648673-57648720	
ZNF702P	79986	A_17_P10996609	0.037	1.9	chr19:58188816-58188860	Other
ZNF763	284390	A_17_P31500433	0.046	1.9	chr19:11937303-11937347	Other
ZNF793	390927	A_17_P17136071	0.036	2.3	chr19:42689868-42689912	Other
ZSCAN1	284312	A_17_P17165786	0.039	1.9	chr19:63237712-63237756	Other

Table S2. Top 100 differentially expressed genes (under- and overexpressed) in PeCa.

DOWN-EXPRESSED				OVEREXPRESSED			
GENE	***FC***	***GENE***	***FC***	***Gene***	***FC***	***GENE***	***FC***
ANGPTL7	-347.6	*GRIN2A*	-37.2	*MMP12*	89.91	*ERCC6L*	13.20
ATP1A2	-246.4	*NTF3*	-35.8	*MMP10*	52.53	*BUB1B*	13.17
FRZB	-194.5	*PDK4*	-35.7	*IL1B*	51.42	*CA2*	13.13
AG2	-137.7	*CFD*	-35.7	*MMP1*	51.09	*LAMP3*	13.10
MYOC	-137.1	*GALNTL1*	-35.6	*IL1A*	42.84	*HAS3*	13.08
CNN1	-98.9	*MYH11*	-34.9	*CXCL1*	38.84	*TNF*	12.89
CPAMD8	-97.5	*KCNE4*	-34.2	*EREG*	27.23	*RAD51*	12.79
CHRDL1	-89.1	*LMOD1*	-34.2	*CASC5*	26.23	*KRT75*	12.77
PCDH20	-89.0	*GPM6A*	-34.1	*AIM2*	26.07	*CYP27B1*	12.70
AOC3	-82.8	*FRY*	-33.6	*RGS20*	26.02	*LMNB1*	12.62
MYRIP	-75.5	*PTCHD1*	-33.5	*APOBEC3A*	24.40	*MUCL1*	12.45
PCOLCE2	-70.2	*CNR1*	-33.3	*CTSL2*	24.28	*TCHH*	12.36
CILP	-69.6	*KCNB1*	-33.3	*SKA3*	23.16	*OVOL1*	12.34
FAM150B	-69.6	*ZBTB16*	-33.1	*CEP55*	23.10	*ASPM*	12.24
OLFM4	-66.2	*SCARA5*	-33.0	*NETO2*	21.82	*OASL*	12.14
PLCXD3	-65.5	*NUDT10*	-32.8	*BUB1*	20.90	*KYNU*	12.11
VIT	-64.5	*NLGN1*	-32.7	*E2F7*	20.72	*TOP2A*	12.11
ACTG2	-62.8	*CRLF1*	-31.9	*SLC28A3*	19.95	*KIF18A*	12.05
RGS5	-61.9	*CLEC3B*	-31.4	*EPCAM*	19.86	*GJB2*	12.01
EDN3	-60.3	*TMEM47*	-31.2	*IL12RB2*	19.78	*KIF20A*	11.89
RCAN2	-60.3	*XKR4*	-30.9	*CENPA*	19.55	*VSNL1*	11.86
PRDM16	-59.3	*PRUNE2*	-30.8	*CXCL13*	19.47	*SERPINB5*	11.85
ADRA1B	-57.4	*AR*	-30.5	*CKAP2L*	18.72	*C1ORF135*	11.84
PLP1	-54.0	*BEX1*	-29.7	*KRT16*	18.68	*UHRF1*	11.82
SLC17A8	-52.9	*MAOB*	-29.3	*KIAA0101*	18.38	*FAM64A*	11.77
ITGA8	-52.7	*TMOD1*	-29.2	*TTK*	17.91	*TPX2*	11.73
LONRF2	-52.6	*SLITRK4*	-29.1	*KIAA1199*	17.44	*MELK*	11.68
SYNM	-52.1	*SRD5A2*	-29.0	*CHEK1*	17.43	*KRT17*	11.45
MYOT	-51.5	*ITIH3*	-29.0	*PKP1*	16.91	*HMGB3L1*	11.44
SPARCL1	-49.8	*RAI2*	-28.4	*MMP9*	16.88	*MMP7*	11.38
DES	-49.7	*HBB*	-28.4	*SHCBP1*	16.59	*CCNA2*	11.35
IPO9	-49.7	*PLCB4*	-28.2	*MAGEA1*	16.14	*SLC7A11*	11.07
LPHN3	-49.6	*THBS4*	-28.0	*NCAPG*	15.95	*KRT33A*	10.95
ARHGAP20	-49.3	*MYLK*	-27.5	*IFNE*	15.77	*NUF2*	10.93
SCRG1	-48.8	*CCDC85A*	-27.4	*IL11*	15.27	*CENPF*	10.86
CKMT2	-47.5	*C8ORF22*	-27.2	*BNC1*	15.12	*MCM10*	10.85
COL21A1	-47.4	*PNMT*	-27.2	*IL1F5*	15.00	*DTL*	10.82
PDLIM3	-44.6	*LOC388630*	-26.4	*EPGN*	14.80	*PBK*	10.81
GNAZ	-44.2	*PLAC9*	-26.3	*AREG*	14.35	*CDCA5*	10.78
ARHGAP6	-43.6	*FOXF1*	-25.9	*MMP13*	14.19	*UBE2T*	10.78
TYRP1	-42.8	*PKNOX2*	-25.8	*CYP24A1*	14.18	*GAL*	10.77
PDZRN4	-41.6	*C2ORF40*	-25.7	*SPC25*	14.17	*CDC6*	10.77
FHL1	-41.3	*HSPB7*	-25.6	*SPRR2D*	13.97	*CDCA8*	10.70
RNF112	-41.0	*SORBS1*	-25.6	*LCE3D*	13.93	*CCNB1*	10.69
PPP1R1A	-40.7	*C10ORF10*	-25.6	*PPIF*	13.86	*C19ORF21*	10.69
RERGL	-40.7	*C8ORF84*	-25.5	*DLGAP5*	13.62	*FAM111B*	10.60
PLN	-39.8	*MAL*	-25.3	*HMMR*	13.55	*AGMAT*	10.59
TMEM100	-39.0	*MAGI2*	-25.1	*CCL20*	13.50	*TNNT1*	10.43
PPP1R3C	-38.7	*FHL5*	-25.1	*CDK1*	13.41	*B3GNT5*	10.38
CASQ2	-38.2	*HSPB2*	-25.0	*FOXM1*	13.21	*KRT16P3*	10.35

FC – fold change

Table S3. Integrative analysis for transcriptome data and methylation profile, according to HPV status.

	HPV +			HPV -	
Gene	Fold Expression	Fold Methylation	Gene	Fold Expression	Fold Methylation
PRR16	-1.29	1.71	*ADRA1B*	-1.17	1.44
FSCN1	-1.26	1.86	*SMOC1*	-1.21	1.53
FZD5	-1.09	1.70	*TNFSF14*	-1.94	2.18
TRPM2	-3.09	2.01	*EBF2*	-1.16	1.78
HN1	-1.48	1.41	*BLM*	-1.03	1.55
PRAME	-4.90	1.70	*TBX3*	-1.18	1.82
CD70	-1.22	1.37	*VAC14*	-1.09	1.42
PKP3	-1.21	1.72	*PLEKHA4*	-1.08	2.09
BCL11B	-1.51	1.77	*MMP9*	-1.61	1.82
TERT	-1.08	1.65	*NOG*	-1.04	1.80
TOLLIP	-1.31	2.17	*MLXIPL*	-1.29	1.71
KLHDC7B	-1.32	1.63	*KLHL17*	-1.65	1.91
TOX2	-1.26	1.95	*NRP1*	-1.10	1.82
LRRK2	-1.18	1.86	*TMEM130*	-2.00	1.86
WDR4	-1.21	2.04	*RTN4R*	-1.17	1.58
RUNX1	-1.88	1.89	*GJA3*	-1.37	1.66
PLEKHA4	-1.08	2.09	*NRN1*	-1.19	2.33
ENPP1	-1.20	1.71	*FAM83H*	-2.16	1.67
C15orf48	-1.98	1.52	*GAD1*	-2.01	1.73
KRT86	-1.57	1.57	*SLC25A29*	-1.23	1.59
PLCH2	-1.63	1.90	*SIX1*	-1.18	1.77
ARID3A	-1.31	1.68	*CA3*	-1.11	1.63
TNFSF14	-1.94	2.18	*SNIP*	-1.14	1.81
GNA14	-1.10	2.06	*LAMA1*	-1.01	1.82
GJA3	-1.37	1.66	*C1orf144*	-1.17	1.43
EGFL6	-1.24	1.65	*PNMA3*	-1.26	1.75
KLHL17	-1.65	1.91	*CIDEA*	-2.05	1.69
PITX1	-1.40	1.70	*KREMEN2*	-1.22	1.83
MMP9	-1.61	1.82	*OSR2*	1.04	-1.58
PTPRZ1	-1.23	1.52	*SLC8A1*	-1.88	1.76
RTN4R	-1.17	1.58	*OLFML2A*	-1.04	1.83
TMEFF2	-1.17	1.60	*PLLP*	-1.05	1.45
SIRPA	-1.02	1.71	*MAL*	-1.87	1.74
FLJ40504	-1.68	1.74	*FSCN1*	-1.26	1.86
GAD1	-2.01	1.73	*PHF21B*	-1.38	1.75
EML2	-1.35	1.46	*SLC2A13*	-1.29	1.84
GATA2	-1.03	1.72	*PKP3*	-1.21	1.72
SLC1A3	-1.72	1.81	*FBXL7*	-1.03	1.79
UNC13A	-1.13	1.61	*FLJ40504*	-1.68	1.74
SLC25A29	-1.23	1.59	*ONECUT2*	-1.15	1.92
TUBB3	-1.10	2.13	*TOX*	-1.53	1.57
LAMA1	-1.01	1.82	*IGF2BP3*	-3.81	1.64
C6orf204	-1.24	1.65	*CHRM2*	-1.69	1.66
PELI3	-1.05	1.82	*PXDNL*	-4.76	2.00
ERBB4	-1.08	1.80	*WDR4*	-1.21	2.04
DCBLD1	-1.62	1.55	*LOC389634*	-1.03	1.53
CHRM2	-1.69	1.66	*PRKCZ*	-1.12	1.69
ABCB1	-1.06	1.97	*NSUN7*	-1.09	1.68
NRP1	-1.10	1.82	*SLC9A7*	-2.20	1.34
LMNB2	-1.09	1.82	*PRDM5*	-1.11	1.66
RBM11	-1.01	1.97	*KCNMA1*	-1.92	1.56
PHF21B	-1.38	1.75	*CD70*	-1.22	1.37
SNX10	-2.56	1.62	*TOX2*	-1.26	1.95
DIP2C	-1.11	1.83	*FZD5*	-1.09	1.70
PCDHB12	-1.19	1.71	*PRAME*	-4.90	1.70
PPP1R14A	-1.22	1.61	*TUBB3*	-1.10	2.13
PCDHB7	-1.08	1.84	*ERBB4*	-1.08	1.80
CLYBL	-1.20	1.52	*HS3ST2*	-1.28	1.77
FLI1	-1.11	1.77	*RPS6KA4*	-1.12	1.74
PCSK6	-1.26	1.84	*TMEFF2*	-1.17	1.60
PRKAG2	-1.26	1.52			
TAC1	-1.02	1.51			
MAL	-1.87	1.74			
NBLA00301	-1.08	1.70			
CXXC4	-1.25	2.30			

Table S4. Top ranked pathways obtained from the 122 differentially methylated probes for the Grade 3 tumors. KOBAS and GSEA software were used for the enrichment analysis.

Enrichment for Pathway Identification	Database	Input number	p-value Kobas	p-value GSEA
ECM-receptor interaction	KEGG Pathway	5	8.7E-05	3.7 E-05
Cell migration	Reactome	8	3.9 E-04	6.2 E-05
Developmental Biology	Reactome	9	1.2 E-03	2.1 E-05
Arf6 signaling events	PID	3	2.3 E-03	6.4 E-05
L1CAM interactions	Reactome	4	2.6 E-0-3	4.1 E-05

Table S5. Comparison between gene methylation pattern and transcript levels with clinical and pathologic parameters.

Gene	*Low Methylation levels*	*High expression levels*	*Prognosis*
NKX2.3	0.014[a]		*Shorter disease free survival*
BDNF	0.018[a]		*Shorter disease free survival*
	0.035[b]		*Lymph nodes metastasis*
FOXA1		0.033[b]	*Perineural invasion*
LIN28A	0.023[b]		*Clinical stages III and VI*
CDX2	0.041[b]		*Clinical stages III and VI*
OTX2		0.040[b]	*Angiolymphatic invasion*

[a]Log rank test - univariate analysis
[b]Mann -Whitney test. - univariate analysis

Table S6. Top ranked functions and canonical pathways as identified by *in silico* analysis. Under- and overexpressed and hypermethylated genes were used as to input in the IPA, KOBAS and GSEA software.

Transcriptome Analysis		
**Functions Annotation**	**Number of Genes**	**p-value**
Cellular movement	540	1.28E-36[#]
Cell migration	493	4.98E-35[+]
Embryonic development, cell development	305	5.2E-33[+]
Angiogenesis	271	4.15E-29[#]
Cellular growth and proliferation	802	2.29E-27[+]
Cell cycle	154	3.12E-21[+]
Epithelial/mesenchymal transition	55	2.39E-7[+]
Extracellular matrix	33	2.79E-7[+]
Stem cell proliferation	46	3.75E-7[+]
Cell-to-cell Signaling and Interaction	82	1.27E-7[+]
**Canonical Pathways**		
Molecular mechanisms of cancer	81	2.09E-26[+]
Calcium signaling pathway	53	8.79E-22[#]
MAPK signaling pathway	63	9.14E-20[+]
Cell cycle, Mitotic	90	2.81E-33[+]

Genome-wide methylation		
**Functions Annotation**	**Number of Genes**	**p-value**
Embryonic development, Organismal development	78	5.2E-33[+]
DNA binding	72	2.61E-21[+]
Cell development	25	2.14E-20[+]
Gene expression	83	4.36E-19[#]
Cell-to-cell Signaling and Interaction	29	5.62E-11[+]
Ion transport	4	6.94E-04[#]
**Canonical Pathways**		
Transcriptional regulatory network in embryonic stem cells	9	2.45E-09[&]
MAPK signaling pathway	7	2.23E-06[#]
Wnt/β-catenin Signaling	8	2.13E-03[&]

[+] IPA Function annotation or Canonical Pathways were confirmed by KOBAS and GSEA software. [#] IPA Function annotation or Canonical Pathways were confirmed by KOBAS software. [&] Canonical Pathways obtained only from IPA software.

Table S7. Genes provided from the upstream regulator analysis (Ingenuity Pathways Analysis software - IPA). Genes in the first column were altered (under- or overexpressed) in PeCa and are known to regulate several genes identified as differentially expressed in the transcriptome analysis.

Upstream Regulator	*Log Ratio*	*Molecule Type*	*Predicted Activation State*	*Z-score*	*Drug(s)*
SRF	Down-expressed	Transcription regulator	Inhibited	-4.055	
SIRT1	Down-expressed	Transcription regulator	Inhibited	-4.003	
NUPR1	Down-expressed	Transcription regulator	Inhibited	-3.791	
MEF2C	Down-expressed	Transcription regulator	Inhibited	-3.134	
EPAS1	Down-expressed	Transcription regulator	Inhibited	-3.079	
ERG	Down-expressed	Transcription regulator	Inhibited	-2.910	
ARNT2	Down-expressed	Transcription regulator	Inhibited	-2.832	
MITF	Down-expressed	Transcription regulator	Inhibited	-2.614	
ZEB1	Down-expressed	Transcription regulator	Inhibited	-2.583	
SIM1	Down-expressed	Transcription regulator	Inhibited	-2.545	
TBX5	Down-expressed	Transcription regulator	Inhibited	-2.499	
ATF3	Down-expressed	Transcription regulator	Inhibited	-2.495	
TGFB3	Down-expressed	Growth factor	Inhibited	-2.494	
NOTCH4	Down-expressed	Transcription regulator	Inhibited	-2.378	
ESR1	Down-expressed	Ligand-dependent nuclear receptor	Inhibited	-2.367	17-alpha-ethinylestradiol, fulvestrant, tamoxifen
NPPA	Down-expressed	Other	Inhibited	-2.051	
MERTK	Down-expressed	Kinase	Inhibited	-2.011	
FOXM1	Overexpressed	Transcription regulator	Activated	2.004	
TLR6	Overexpressed	Transmembrane receptor	Activated	2.034	
S100A9	Overexpressed	Other	Activated	2.044	
OSM	Overexpressed	Cytokine	Activated	2.089	
F2RL1	Overexpressed	G-protein coupled Receptor	Activated	2.183	
PTGES	Overexpressed	Enzyme	Activated	2.195	
PIK3CD	Overexpressed	Kinase	Activated	2.204	SF 1126, PX-866, NVP-BEZ235, BKM120
PLAU	Overexpressed	Peptidase	Activated	2.476	
EZH2	Overexpressed	Transcription regulator	Activated	2.503	
CASP1	Overexpressed	Peptidase	Activated	2.589	
FGF1	Overexpressed	Growth factor	Activated	2.672	pentosan polysulfate
E2F2	Overexpressed	Transcription regulator	Activated	2.688	
TFDP1	Overexpressed	Transcription regulator	Activated	2.804	
CCL5	Overexpressed	Cytokine	Activated	2.940	
S100A7	Overexpressed	Other	Activated	2.985	
STAT1	Overexpressed	Transcription regulator	Activated	3.009	
IL1A	Overexpressed	Cytokine	Activated	3.158	IL-1 trap
E2F1	Overexpressed	Transcription regulator	Activated	3.579	
IL1B	Overexpressed	Cytokine	Activated	4.011	IL-1 trap, canakinumab
TP63	Overexpressed	Transcription regulator	Activated	4.132	
IFNG	Overexpressed	Cytokine	Activated	4.839	
TNF	Overexpressed	Cytokine	Activated	6.014	adalimumab, etanercept, infliximab, golimumab
CSF2	Overexpressed	Cytokine	Activated	6.342	

Table S8. Clinical and pathological characteristics of the penile carcinomas patients.

**Variable**	**Number of Patients (%) N=44**
Age (years)	
≤ 45	10 (22.7)
> 45	34 (77.3)
Average age	57.1 years
Min age	24 years
Max age	92 years
Smoking usage	
Yes	12 (27.3)
No	28 (63.6)
NA	4 (9.1)
Alcohol consumption	
Yes	7 (15.9)
No	30 (68.2)
NA	7 (15.9)
Histological type[&]	
SCC usual/classical	41 (93.2)
SCC mixed*	3 (6.8)
Clinical Stage	
I	12 (27.3)
II	13 (29.5)
III	13 (29.5)
IV	6 (13.7)
Histological grade	
I	9 (20.5)
II	22 (50.0)
III	13 (29.5)
Lymph node metastasis	
Yes	12 (27.3)
No	14 (31.8)
NL	18 (40.9)
HPV infection	
High risk (16/18/31/33)	17 (38.6)
No	27 (61.4)
Perineural invasion	
Yes	10 (22.7)
No	34 (77.3)
Type of surgery	
Partial penectomy	37 (84.1)
Total penectomy	7 (15.9)

NA: data not available
NL: Lymphadenectomy was not performed
SCC: squamous cell carcinoma
[&] Histopathological classification was performed according to the International Union Against Cancer (TNM, 2002). SCC mixed* (one SCC usual/sarcomatoid and two SCC usual/papillary)

Table S9. Primer sequences and probe properties for those evaluated by pyrosequencing.

Gene symbol	Primer sequences	Amplicon lenght	Number of CpG
CD133	F - 5´ TGTTAGAGAGAAGGGGTTTTGGAG 3´<br>R - 5´ [B]ACCCCTAACTTAAAACACCTTCCC 3´<br>Seq - 5´ TAGTTTTTTATTTAG 3´<br>Py - 5´ **CG**ATAGT**CGCG**GGTTTT**CGCG**T 3´	280 bp	5
FOXA1	F - 5´ TTTAGTTGTGGGGAGGATGG 3´<br>R - 5´ [B]CACCCTACAATCCTCACTAC 3´<br>Seq - 5´ TGTTTGTGTTTAAGA 3´<br>Py - 5´ **CG**GGTTTG**CG**ATAGTTTGGGG**CG**GTTTAGGT**CGCG** 3´	191 bp	5
LIN28A	F - 5´ GGGGTTAGGAATTTTGGTGTT 3´<br>R - 5´ [B]CCAACCCCACAAATAAACAAC 3´<br>Seq - 5´ GGGTATTATTGTTT 3´<br>Py - 5´ **CG**GTGTA**CG**TTAGGGGAA**CGCG**GGAGG**CG** 3´	237 bp	5
TWIST1	F - 5´ AGAGTTTGGGGTTTAGGAGGT 3´<br>R - 5´ [B]TACCCTCCAAATCCCCTCT 3´<br>Seq - 5´ TTTGTAAATGTT 3´<br>5´ Py - **CG**AGTTTTTT**CG**GAGGAAAGAT**CG**TTAGGG**CG**A**CG** 3´	293 bp	5
MEIS1	F - 5´ GAATTGGATAATTTGGTAAGAG 3´<br>R - 5´ [B]TACTACCTTCCTTACACCTTTC 3´<br>Seq - 5´ AATGTTTTTGTATA 3´<br>Py - 5´ **CG**GATAATTGGTT**CG**GGATAAGATTT**CG**G 3´	425 bp	3
WIT1	F - 5´ GGTTATTGGGGAAAGAGGTGGT 3´<br>R - 5´ [B]TTCACCCCTCCTCCCCAA 3´<br>Seq - 5´ GTATTAGTTATAAGT 3´<br>Py - 5´ **CG**GG**CG**TATTT**CG**AT**CG**G 3´	356 bp	4
PAX7	F - 5´ AGAGGGGTAYGGTAATGTTTAAA 3´<br>R - 5´ [B]AACCTTTTCTCCAACCAAAAC 3´<br>Seq - 5´ AGAGGGGTAYGGTAATGTTTAAA 3´<br>Py - 5´ **CG**AGAAGAAAGAGTTGATTTTGTAAAG**CG** 3´	137 bp	2
HOXA3	F - 5´ GGTTAGGGGAGAGGTAAGAG 3´<br>R - 5´ [B]CCAAACCAAACCRAAAACC 3´<br>Seq - 5´ ATTTTTTTTGAAT 3´<br>Py - 5´ **CG**AGATTTAAGTTTGTAGTTATAAATTAT**CG**AGA**CG** 3´	269 bp	3
ONECUT1	F - 5´ GGAGGGGGTTATAGTAGTTAG 3´<br>R - 5´ [B]CAAATTACCTACTCCAAAACTC 3´<br>Seq - 5´ TGTTAGAGGTATTTG 3´<br>Py - 5´ **CG**AGGAATATTTG**CG**TG**CG**ATTTTA**CG** 3´	332 bp	4
ONECUT2	F - 5´ GTTTGGGGAGGGGGAGTT 3´<br>R - 5´ [B]CCAACTACCAAATTCCTTCTAATCC 3´<br>Seq - 5´ GTAGTTAGTAGAGAG 3´<br>Py - 5´ **CG**ATAATAATAG**CG**G**CG**G 3´	432 bp	3
LHX5	F - 5´ GAGGGAGTTAGGAGTGAGTT 3´<br>R - 5´ [B]ACTTCTACCCCAAAAAACC 3´<br>Seq - 5´ TGGTGGGTTGGGT 3´<br>Py - 5´ **CG**GG**CG**GGG**CG**GTTTTGG**CG**T 3´	476 bp	4
NKX2.2	F - 5´ GGGTATATGTTAGGATTTTGATA 3´<br>R - 5´ [B]CCTAAAAAAACCCAAATCTC 3´<br>Seq - 5´ GTTAAATTGAATA 3´<br>Py - 5´ **CG**AATTGTGGTTT**CG**GGTA**CG** 3´	430 bp	3

Cont.

Gene symbol	Primer sequences	Amplicon lenght	Number of CpG
NKX2.3	F - 5´ AGGGATTTAGTAGTGGGTTTTTAG 3´<br>R - 5´ [B]TCCTACCCCAACCAAACTC 3´<br>Seq - 5´ AGGGATTTAGTAGTGGGTTTTTAG 3´<br>Py - 5´ **CG**GGTTTTGTTTTGG**CG**T**CG**GGGTTGTAGATAT**CG** 3´	202 bp	4
SOX3	F - 5´ GAGTGGGAATGAGAAGTTATTT 3´<br>R - 5´ [B]ACCCAACATACAAAAATCCC 3´<br>Seq - TATTATTTTTAGTTT 3´<br>Py - 5´ **CG**TGGAAAGGTGGGGAATTAAA**CG** 3´	226 bp	2
SOX14	F - 5´ TTGGTTTGTTYGTGGGGGT 3´<br>R - 5´ [B]AAACRCCRTTCTAATAACCCA 3´<br>Seq - 5´ ATTTTTTGTTGGATTT 3´<br>Py - 5´ **CGCG**TAGTTTAGTT**CG**AG**CG** 3´	207 bp	4
SOX17	F - 5´ GTTGTTTTATTTGGGAGGTG 3´<br>R - [B]ACCCCTAAATAACCAAACAAA 3´<br>Seq - 5´ GATTTGTTTTTTAG 3´<br>Py - 5´ **CG**TTAATT**CG**GATTTGTTTAGGTGTTTA**CG** 3´	369 bp	3
CDX2	F - 5´ TGGGAGTTAGGATTTGGGAGT 3´<br>R - 5´ [B]TCTTTCCCCAACCCTCAAC 3´<br>Seq - 5´ TAGTTTTTAAAGGG 3´<br>Py - 5´ **CG**TTTTG 3´	320 bp	1
RSPO2	F - 5´ GTAGGTGGTGTTGTGGTTTAAGG 3´<br>R - 5´ [B]TCCCCAACTACTCTTTATCTATCCC 3´<br>Seq - 5´ GTTTTAAAGTTAGAAA 3´<br>Py - 5´ **CG**TTT**CG**GTTTTTTTGAGGTTTTAAAGAA**CG** 3´	187 bp	3
OTX2	F - 5´ GTTTTAGTGTGAGTTAGGG 3´<br>R - 5´ [B]ACAATATCTCTCCCTAACAC 3´<br>Seq - 5´ AGGTTAGATGGGAGG 3´<br>Py - 5´ **CG**TGTATTTTTTGGGGAGTT**CG**TAAAGTTTTT**CG** 3´	162 bp	3
BDNF	F - 5´ TAGTAGGGAGGGGGTGG 3´<br>R - 5´ [B]TCATAACTTCATTCAACTCAACC 3´<br>Seq - 5´ GAATATTTGTATG 3´<br>Py - 5´ **CG**T**CG**AAG**CGCG**AA 3´	378 bp	4

F: forward primer; R: reverse primer; Seq: sequencing primer; Py: reference sequence (CGs in bold are the CpG sites analyzed by pyrosequencing); bp: base pair.

Table S10. Primer sequences and transcript properties for those evaluated by RT-qPCR.

Gene symbol	Accession number	Primer sequences	Amplicon length
ACTB*	NM_001101.3	F - 5'TTCCTGGGCATGGAGTC3'<br>R - 5'CAGGTCTTTGCGGATGTC3'	84bp
GAPDH*	NM_002046<br>NM_001256799.1	F - 5'TGCACCACCAACTGCTTAG3'<br>R - 5'GAGGCAGGGATGATGTTC3'	176bp
GUSB*	NM_000181.3	F - 5'GAAAATACGTGGTTGGAGAGCTCATT3'<br>R - 5'CCGAGTGAAGATCCCCTTTTTA3'	101bp
HMBS*	NM_000190.3 NM_001258208.1<br>NM_001258209.1	F - 5'GGCAATGCGGCTGCAA3'<br>R - 5'GGGTACCCACGCGAATCAC3'	64bp
HPRT1*	NM_000194.2	F - 5'TCATTATGCTGAGGATTTGGAAAG3'<br>R - 5'GGCCTCCCATCTCCTTCATC3'	101bp
RPLP0*	NM_001002.3<br>NM_053275.3	F - 5'GGAGACGGATTACACCTTC3'<br>R - 5'TTCAACCTTAGCTGGGG3'	139bp
FOXA1	NM_004496.3	F - 5'AGATGGAAGGGCATGAAACCA3'<br>R - 5'GCCTGAGTTCATGTTGCTGAC3'	95bp
HOXA3	NM_030661.4<br>NM_153631.2	F - 5'ACAAAGCAGAAAACCAGCAGC3'<br>R - 5'GGCACAGGTAGCGGTTGAAG3'	157bp
MEIS1	NM_002398.2	F - 5'GGGAATTAGAGCACCAGGACC3'<br>R - 5'GCTGTCCCATACTCACACCC3'	179bp
RSPO2	NM_178565.4	F - 5'CCATGGAATGTGTGGAAGGATG3'<br>R - 5'TTCTGGTTTCCAGACCCCAT3'	105bp
PAX7	NM_002584.2<br>NM_013945.2 NM_001135254.1	F - 5'GTCAGCAACGGCCTGTCT3'<br>R - 5'TCTCCTGGCTTGATGGAGTC3'	170bp
PROM1/ CD133	NM_006017.2 NM_001145847.1<br>NM_001145848.1<br>NM_001145849.1<br>NM_001145850.1<br>NM_001145851.1<br>NM_001145852.1	F - 5'AGCTGATGTTGAAACTGCTTGAG3'<br>R - 5'CTGGCGTTGCTCCTGGATTT3'	110bp
SOX3	NM_005634.2	F - 5'TATAGCCAGGTGTGCGTGTG3'<br>R - 5'AAGTAGCCCCTCCCCACTAC3'	158bp
SOX14	NM_004189.3	F - 5'GTCGGGTTATGAAGTCAGGAAGT3'<br>R - 5'CAAATGAACAGAGCAACGAGGT3'	136bp
SOX17	NM_022454.3	F - 5'AAGTAGAAGGGGATGTCCAAGT3'<br>R - 5'TTAAACCCAGCGATGCTTGC3'	136bp

Cont.

Gene symbol	Accession number	Primer sequences	Amplicon length
TWIST1	NM_001753.4	F - 5'ACCTAGATGTCATTGTTTCCAGAG3'<br>R - 5'CCACGCCCTGTTTCTTTGAAT3'	143bp
ONECUT1	NM_004498.1	F - 5'CCCAAACCCTGGAGCAAACT3'<br>R - 5'GTTGCCTCTATCCTTCCCATGT3'	150bp
OTX2	NM_021728.3<br>NM_001270525.1<br>NM_001270524.1<br>NM_001270523.1<br>NM_172337.2	F - 5'CTTCATGCGAGAGGAGGTGG3'<br>R - 5'TCTGCTGTTGTTGCTGTTGT3'	110bp

F: forward primer; R: reverse primer; bp: base pair.
*: Candidates for reference transcripts.
